# Inhibition of the chemokine receptors CXCR1 and CXCR2 synergizes with docetaxel for effective tumor control and remodeling of the immune microenvironment of HPV-negative head and neck cancer models

**DOI:** 10.1186/s13046-024-03240-3

**Published:** 2024-12-05

**Authors:** Lucas A. Horn, Hanne Lind, Kristen Fousek, Haiyan Qin, Nika Rajabian, Shantel Angstadt, Nicole Hsiao-Sanchez, Miriam M. Medina-Enriquez, Marcus D. Kelly, Clint T. Allen, Sarah M. Hammoudeh, Roberto Weigert, Dean Y. Maeda, John A. Zebala, Claudia Palena

**Affiliations:** 1 Center for Immuno-Oncology, Center for Cancer Research, National Cancer Institute, National Institutes of Health, Bethesda, MD USA; 2 Surgical Oncology Program, Center for Cancer Research, National Cancer Institute, National Institutes of Health, Bethesda, MD USA; 3 Laboratory of Cellular and Molecular Biology, Center for Cancer Research, National Cancer Institute, National Institutes of Health, Bethesda, MD USA; 4 Syntrix Pharmaceuticals, Auburn, WA USA

**Keywords:** HNSCC, CXCR1, CXCR2, IL-8, Docetaxel, Immunotherapy

## Abstract

**Background:**

Relapsed head and neck squamous cell carcinoma (HNSCC) unrelated to HPV infection carries a poor prognosis. Novel approaches are needed to improve the clinical outcome and prolong survival in this patient population which has poor long-term responses to immune checkpoint blockade. This study evaluated the chemokine receptors CXCR1 and CXCR2 as potential novel targets for the treatment of HPV-negative HNSCC.

**Methods:**

Expression of IL-8, CXCR1, and CXCR2 was investigated in HNSCC tissues and human cell line models. Inhibition of CXCR1/2 with the clinical stage, small molecule inhibitor, SX-682, was evaluated in vitro and in vivo using human xenografts and murine models of HNSCC, both as a monotherapy and in combination with the taxane chemotherapy, docetaxel.

**Results:**

High levels of IL-8, CXCR1, and CXCR2 expression were observed in HPV-negative compared to HPV-positive HNSCC tumors or cell lines. Treatment of HPV-negative HNSCC cell lines in vitro with SX-682 sensitized the tumor cells to the cytotoxic activity of docetaxel. In vivo, treatment of HNSCC xenograft models with the combination of SX-682 plus docetaxel led to strong anti-tumor control resulting in tumor cures. This phenomenon was associated with an increase of microRNA-200c and a decreased expression of its target, tubulin beta-3, a protein involved in resistance to microtubule-targeting chemotherapies. In vivo treatment of a murine syngeneic model of HNSCC with SX-682 plus docetaxel led to potent anti-tumor efficacy through a simultaneous decrease in suppressive CXCR2^+^ polymorphonuclear, myeloid-derived suppressor cells and an increase in cytotoxic CD8^+^ T cells in the combination therapy treated tumors compared to controls.

**Conclusions:**

This study reports, for the first time, mechanistic findings through which the combination of CXCR1/2 inhibition and docetaxel chemotherapy exhibits synergy in models of HPV-negative HNSCC. These findings provide rationale for the use of this novel combination approach to treat HPV-negative HNSCC patients and for future combination studies of CXCR1/2 inhibition, docetaxel, and immune-based therapies.

**Supplementary Information:**

The online version contains supplementary material available at 10.1186/s13046-024-03240-3.

## Background

Head and neck squamous cell carcinomas (HNSCC) are a group of heterogeneous tumors that originate in the epithelial cells of the mucosal lining of the oral cavity, pharynx, and larynx [1]. While most cases of HNSCC are associated with exposure to tobacco-derived carcinogens and alcohol consumption (HPV-negative HNSCC), a subset of cases arising from the oropharynx are associated with high-risk human papillomavirus (HPV) infection (HPV-positive HNSCC) [1]. At the time of diagnosis, a large proportion of patients with HPV-negative and HPV-positive HNSCC present with locally advanced disease requiring combined treatment approaches, including surgical resection, radiation, and chemotherapy [2]. For HPV-positive HNSCC, cure rates are high, and prognosis is favorable following these standard of care treatments; however, for HPV-negative HNSCC, half of patients treated with these standard of care approaches will relapse locoregionally or distally within 2 years. For patients with relapsed disease, targeting the programmed cell death 1 receptor (PD-1) with the antibody pembrolizumab is currently approved as first-line treatment [3–5]. Although pembrolizumab can lead to durable responses in this setting, overall response rates are low. Novel therapeutic approaches that aim to improve the clinical outcome of patients with anti-PD-1 refractory or relapsed HNSCC are needed.

Our laboratory and others have previously demonstrated a role for the chemokine interleukin-8 (IL-8, CXCL8) in mechanisms of tumor progression in several tumor types, including as a driver of resistance to chemotherapy [6], EGFR-targeted therapy [7], and immunotherapy [8–11]. IL-8 is a chemokine of the CXCL family that binds to two G-protein coupled receptors, CXCR1 and CXCR2, expressed on myeloid cells, endothelium, and cancer cells. In addition to IL-8, CXCR1/2 bind six other redundant chemokines of the same family, CXCL1/2/3/5/6/7 [11]. IL-8 in particular has been shown to facilitate the recruitment of polymorphonuclear myeloid-derived suppressor cells (PMN-MDSC) to the tumor microenvironment (TME) [8, 12] while also driving tumor cell plasticity in the context of an epithelial-mesenchymal transition (EMT). The process of EMT involves the loss of cell-to-cell contacts and cell polarity and the gain of mesenchymal protein expression, which ultimately leads to increased tumor cell migration, invasiveness, and resistance to cell death [13]. IL-8 is elevated in the serum of patients with recurrent or metastatic HNSCC compared to the levels observed in newly diagnosed HNSCC patients or healthy donors [14]. In addition, high expression of IL-8 in the primary tumor has been associated with poor overall survival in HNSCC [15, 16]. Although these studies indicated a potential role for IL-8 in HNSCC progression, the association of IL-8 with HPV status was not previously evaluated.

In the present work, the potential link between CXCR1/2 signaling and response to chemotherapy in HNSCC has been investigated. Analysis of HNSCC tissues demonstrated that IL-8 and the CXCR1/2 receptors are expressed at higher levels in tumors compared with normal tissues. Furthermore, analysis of patient data in The Cancer Genome Atlas (TCGA) database and characterization of various HNSCC cell line models revealed that expression of IL-8 and CXCR1/2 receptors are significantly higher in HPV-negative HNSCC compared with HPV-positive cases. Inhibition of CXCR1 and CXCR2 signaling via SX-682, a small molecule inhibitor currently under evaluation in the clinic, allowed several human and murine HPV-negative HNSCC models to be sensitized to the cytotoxic activity of docetaxel in vitro and in vivo. This phenomenon was associated with a decrease of tubulin beta-3, a protein involved in conferring resistance to microtubule-targeting chemotherapies. Modulation of the immune compartment in a syngeneic murine model of HPV-negative HNSCC was also observed following combined treatment with docetaxel and SX-682, providing rationale for future combination studies with immune-based therapies.

## Methods

### Cell lines and culture

The human HNSCC cell lines UM-SCC-1, UM-SCC-9, UM-SCC-11A, UM-SCC-11B, UM-SCC-22A, UM-SCC-22B, UM-SCC-47, UM-SCC-74A, and UM-SCC-74B were obtained from Drs. T. E. Carey, M. E. Prince, and C. R. Bradford (University of Michigan, Ann Arbor, MI), and UPCI-SCC-90, UPCI-SCC-152, and UPCI-SCC-154 cells were obtained from Dr. R. Ferris (University of Pittsburgh, Pittsburgh, PA). Cells were cultured in Eagle’s Minimum Essential Medium with 1.5 g/L sodium bicarbonate, non-essential amino acids, L-glutamine, and sodium pyruvate (Corning, Manassas, VA), supplemented with 10% Heat Inactivated Fetal Bovine Serum (FBS; Omega Scientific Inc, Tarzana, CA) and 1X antibiotic/antimycotic solution (Gibco, Billings, MT). Mouse oral cancer-1 (MOC1) cells were obtained from Dr. Ravindra Uppaluri (Dana-Farber Cancer Institute, Boston, MA). Cells were cultured in IMDM/F12 (2:1) medium with 10% FBS, 1% antibiotic–antimycotic (Gibco), 5 ng/mL EGF (ThermoFisher Scientific, Waltham, MA), 400 ng/mL hydrocortisone, and 5 μg/mL insulin. Cell lines were determined to be mycoplasma free by using a MycoAlert Mycoplasma Detection Kit (Lonza, Basel, Switzerland) and used at low passage number from the date received. The identities of UM-SCC-11B, UM-SCC-22B, and UM-SCC-74B were verified through STR analysis (Bio-Synthesis Inc, Lewisville, TX).

### Reagents

The CXCR1/2 small molecule inhibitor, SX-682, was obtained under a Cooperative Research and Development Agreement (CRADA) with Syntrix Pharmaceuticals Inc. (Auburn, WA). Docetaxel (Winthrop, Bridgewater, NJ) and cisplatin (Fresenius Kabi, Lake Zurich, IL) were obtained from the NIH Division of Veterinary Resources Pharmacy.

### RNA in situ hybridization and immunofluorescence

Head and neck cancer tissue arrays containing 70 cases of squamous cell carcinoma and 10 cases of normal tissue (XHN080-01), 60 cases of squamous cell carcinoma with p16 scoring (HN601d), and 78 cases of squamous cell carcinoma and 3 normal head & neck tissues (HN1310) were purchased from US Biolab (Rockville, MD) and TissueArray.com (Derwood, MD). Xenograft and allograft tumor tissues were fixed in Z-fix (Anatech, Battle Creek, Michigan, USA), embedded in paraffin, sectioned, and mounted onto glass slides. Cultured tumor cells were grown in BioCoat Poly-D-Lysine 8-well culture slides (Corning), fixed in 3% paraformaldehyde (Electron Microscopy Sciences, Hatfield, Pennsylvania, USA) for 10 min, and washed with PBS prior to staining. In situ hybridization for detection of IL-8, CXCR1, CXCR2, CXCL1, and CXCL2 mRNA was performed using the RNAscope technology (Advanced Cell Diagnostics, Newark, CA) per the manufacturer’s protocol. Where indicated, RNAscope probes were mixed in equal parts and subsequently used for simultaneous detection of CXCR1 and CXCR2. In some experiments, tissues were stained by antibody immunofluorescence-based detection independently or after RNA in situ hybridization. After deparaffinization and rehydration or RNA in situ hybridization, antigen retrieval was performed by microwaving in pH6 buffer (Akoya Biosciences, Marlborough, MA), followed by blocking with BLOXALL Solution (Vector Laboratories, Burlingame, CA). Primary antibodies against pan-cytokeratin (mouse, polyclonal, catalog ab9377, 1:200 dilution, Abcam, Cambridge, UK), pan-cytokeratin (human, clone AE1/AE3, catalog sc-81714, 1:100 dilution, Santa Cruz Biotechnology, Dallas, TX), anti-CD45 (human; clone HI30, catalog 14–0459-82, 1:100 dilution, ThermoFisher Scientific), anti-CXCR1 (clone MM0221-7D22, catalog ab89251, 1:100 dilution, Abcam), anti-CXCR2 (polyclonal, catalog 20634–1-AP, 1:100 dilution, Proteintech, Rosemont, IL), anti-CD45 (mouse; clone D3F8Q, catalog 70257, 1:200, Cell Signaling, Danvers, MA), anti-HLA Class 1 ABC (clone EMR805, catalog ab70328, 1:1000 dilution, Abcam), and tubulin beta-3 (clone 2G10, catalog MA1-118, 1:100 dilution, ThermoFisher Scientific) were diluted in Renaissance Background Reducing Buffer (BioCare Medical, Pacheco, CA) and incubated for 60 min at room temperature. ImmPress-HRP species-specific IgG Polymer reagents (Vector Laboratories) were used as secondary antibodies; the Opal 4-Color Manual IHC Kit (Akoya Biosciences) was used for signal detection, according to the manufacturer’s protocol. Slides were counterstained with DAPI (Invitrogen, Waltham, MA) and mounted using ProLong Diamond mountant (Invitrogen). Hematoxylin and eosin (H&E) staining was performed by American HistoLabs Inc (Gaithersburg, MD). Slide scanning and image capturing were performed on an Axio Scan.Z1 for tissues or an Axio Observer 7 for cells using Zen Blue software (Zeiss). IL-8 and CXCR1/2 mRNA expression levels were interpreted and scored by two independent scientists; epithelial cells (marked by pan-cytokeratin expression) and the remaining stroma were scored separately. Normal epithelium and tumor cells were scored as positive when > 5% of cytokeratin-positive cells in the tissue core showed positive mRNA signal. The stroma was scored as positive when > 5% of stromal cells (cytokeratin-negative) demonstrated positive mRNA signal. For image quantification, 5–6 equal sized regions of interest (ROI) without obvious signs of necrosis were randomly selected per tumor and analyzed for mean fluorescence intensity (MFI) for each marker of interest.

### Analysis of patient data from the TCGA

RNAseq data from the TCGA head and neck cancer database was downloaded and analyzed using the NIH Integrated Data Analysis Platform (NIDAP). For this analysis, only tumor samples with known HPV status were included. In total, 90 cases of HPV-positive and 410 cases of HPV-negative primary HNSCC tumors were included in the analysis. Differentially expressed genes (DEG) were identified and graphed on a volcano plot. The top 500 up- and downregulated genes by t statistic were analyzed using the l2p package (https://github.com/ccbr/l2p) pathway analysis (REACTOME, HALLMARK). Genes or gene sets with an adjusted *P* value of 0.05 or less were considered statistically significant. Significance of differential expression of individual genes across groups was calculated in GraphPad Prism (GraphPad Software, La Jolla, CA, www.graphpad.com) by Student’s t-test.

### Immunoblot

Tumor cells were grown in complete culture media supplemented with 2% FBS, collected, pelleted, and lysed with RIPA buffer (Santa Cruz) containing 1 mM PMSF (Santa Cruz). A BCA protein assay (ThermoFisher Scientific) was used to quantify protein concentration; proteins (20 µg) were resolved on Bolt 4–12% Bis–Tris Plus Gels (Invitrogen) under reducing conditions and transferred onto nitrocellulose membranes via the iBlot3 Transfer System (Invitrogen). Blotting-Grade Blocker (5%, Bio-Rad, Hercules, CA) in Tris-buffered saline plus 0.1% Tween (KD Medical, Columbia, MD) (TBST) was used to blot membranes for 1 h at room temperature; membranes were probed overnight with primary antibodies at 4 °C, including CXCR1 (clone MM0221-7D22, ab89251, 1:1000, Abcam), CXCR2 (polyclonal, 20634–1-AP, 1:1000, Proteintech), and GAPDH (clone 0411, sc-47724, 1:10,000, Santa Cruz). Membranes were then washed with TBST and probed with secondary antibodies conjugated to IRDye 680 or IRDye 800 (LI-COR Biosciences, Lincoln, Nebraska, USA) and visualized on the Odyssey Infrared Imaging System (LI-COR Biosciences). ImageJ software was used to measure the MFI of bands of interest. A ratio of (MFI of band of interest)/(MFI of corresponding GAPDH band) was calculated.

### Bulk RNA-seq and analysis

Total RNA from control and treated UM-SCC-74B xenografts or murine MOC1 tumors was prepared using a RNeasy Mini Kit (Qiagen, Hilden, Germany) and analyzed on an Agilent TapeStation (Agilent Technologies, Santa Clara, California, USA). Samples with an RNA integrity number (RIN) of greater than 8.0 were sequenced by the Center for Cancer Research Sequencing Facility (Frederick, MD). RNA-Seq FASTQ files were aligned to the corresponding reference genome (GRCh38 and GRCm38, respectively) using STAR [17] and the gene annotation (GENCODE_30 GTF and GENCODE_M21 GTF, respectively) was used to produce raw counts using RSEM [18]. Downstream analysis and visualization were performed within NIDAP using R programs developed by a team of NCI bioinformaticians on the Foundry platform (Palantir Technologies). The RSEM gene counts matrix was imported into the NIDAP platform, where genes were filtered for low counts (< 0.5 cpm), and genes were required to be measured in 3 or more samples for retention in the analysis. Filtered counts were normalized to library size as CPM and normalized by quantile normalization using the limma package [19]. Differentially expressed genes were calculated using limma-Voom [20]. GSEA was performed using fgsea package [21]. Additional pathway enrichment analysis was performed using Fisher's Exact Test as implemented in the l2p package. For comparison of UM-SCC-74B tumors (HALLMARK, GO, C3: Regulatory Target Gene Sets), genes with a *p* value of less than 0.05 and fold-change threshold of 1.2 were used. For comparison of MOC1 tumors (HALLMARK, GO, KEGG), the top 500 up- and downregulated genes by t statistic were used. The DEG output was used for gene set enrichment analysis (GSEA). Both sets of RNAseq data have been deposited in NCBI’s Gene Expression Omnibus under SuperSeries GSE280356.

### Real-time PCR

Total RNA was prepared by using the RNeasy Mini Kit (Qiagen), followed by reverse transcription with the High-Capacity cDNA Reverse Transcription Kit (Applied Biosystems, Foster City, CA), and cDNA was purified with the QIAquick PCR purification kit (Qiagen). cDNA (40 ng) was amplified in triplicate in a LightCycler 96 (Roche, Basel, Switzerland) instrument using the FastStart Essential DNA Probes Master reagent (Roche Diagnostics, Indianapolis, IN) for CXCR2 (Hs01891184_s1, ThermoFisher Scientific) and human GAPDH (4,325,792, Applied Biosystems). Expression of target genes relative to GAPDH was calculated as: 2^−ΔCt^.

### Enzyme-linked immunosorbent assay (ELISA)

Cells were seeded at 5 × 10^5^ cells per well in 6-well plates in culture media supplemented with 2% FBS. After 48 h, supernatants were collected, spun down at 500 g for 5 min and assayed with a human IL-8 Instant ELISA kit (Invitrogen), according to the manufacturer’s instructions. IL-8 concentration was normalized to cell counts at the time of supernatant collection.

### Chemotherapy and siRNA treatment of cell lines

For cisplatin and docetaxel titration experiments, tumor cells were plated in T-75 flasks in complete media plus SX-682 (10 µM) or DMSO at equimolar concentration for 72 h. Cells were harvested, seeded into 96-well plates at 300 cells per well, and allowed to attach; culture media consisted of a 1:1 mix of complete media and tumor-conditioned media (TCM) harvested from the corresponding tumor cell line grown in complete media for 72 h. SX-682 (10 µM) and DMSO were added to their corresponding treatment groups with the addition of cisplatin or docetaxel at indicated concentrations in replicates of 4 wells. Control wells were cultured in the absence of chemotherapies. After 72 h, relative cell viability was evaluated by CellTiter-Glo (Promega, Madison, WI) according to the manufacturer’s instructions; percentage survival was calculated as: 100 x (RLU (chemotherapy) /Average RLU (no chemotherapy). IC50 was calculated using GraphPad Prism.

For tumor cell proliferation experiments, cells were plated in complete culture media supplemented with 2% FBS in black-wall 96-well plates (Greiner Bio-One, Monroe, NC) at a concentration of 4–6 × 10^3^ cells per well. Cells were allowed to attach prior to treatment; SX-682 was added at 1 µM or 2.5 µM. When indicated, cells were transfected with siRNA. Briefly, ON-TARGET plus siRNA SMARTpools (Dharmacon, Lafayette, CO) targeting CXCR1 (L-005646–00–0010) and CXCR2 (L-005647–00–0010) were mixed at an equal ratio and transfected into cells utilizing DharmaFECT 2 transfection reagent (Dharmacon) according to the manufacturer's instructions. After an overnight incubation, docetaxel at indicated concentrations was added to cultures and cells were imaged using the IncuCyte Live-Cell Analysis System (Sartorius, Bohemia, NY) with a 10 × objective every 3–4 h. IncuCyte AI Confluence Analysis was used to quantify confluence percentage within individual wells over time and lines were normalized to a consistent starting point using values from untreated tumor wells.

### Amplification and inhibition of microRNA

Cells were cultured in complete culture media supplemented with recombinant IL-8 (1 ng/mL, R&D Systems, Minneapolis, MN) and either DMSO or SX-682 (5 µM) for 24 h (UM-SCC-11B and UM-SCC-22B) or 48 h (UM-SCC-74B). Micro-RNA was purified using the PureLink miRNA Isolation Kit (Invitrogen), according to the manufacturer’s instructions; next, reverse transcription was performed with a TaqMan MicroRNA Reverse Transcription Kit (Applied Biosystems) in conjunction with miR-specific primers for hsa-mir-200a (ID: 000502), hsa-mir-200b (ID: 002251), hsa-mir-200c (ID: 002300), and snoRNA-135 (ID: 001230) (TaqMan MicroRNA Assay, Applied Biosystems). cDNA was amplified in triplicate using TaqMan Master Mix with no AMP UNG (ThermoFisher Scientific) in an Applied Biosystems 7500 Real-Time PCR System (ThermoFisher Scientific). miRNA expression was normalized to snoRNA-135 by calculating 2^−ΔCt^. The expression of each microRNA in SX-682 treated samples was then normalized to the corresponding untreated control.

For experiments involving inhibition of miRNA in UM-SCC-22B cells, cells were plated in complete culture media supplemented with 2% FBS in 96-well plates at 500–1,000 cells per well and incubated overnight with SX-682 (2.5 μM). For similar experiments using the UM-SCC-74B cell line, cells were plated in 96-well plates at 6,000 cells per well and were allowed to attach for 4 h. Cells were then transfected with either an anti-miR (miRNA Inhibitor, hsa-miR-200c-3p) or an anti-miR negative control (ThermoFisher Scientific) at a concentration of 30 nM or 20 nM, respectively, with Lipofectamine 3000 (Invitrogen), according to the manufacturer’s instructions. After 1-h incubation for UM-SCC-22B or an overnight incubation for UM-SCC-74B, regular culture media containing docetaxel at 0.75 ng/mL or 0.5 ng/mL, respectively, with or without SX-682 was added. UM-SCC-74B experiments were imaged and analyzed using an IncuCyte Live-Cell Analysis System as described above. Following 72-h of incubation, the percentage of UM-SCC-22B cell growth inhibition was determined by CellTiter-Glo as described above.

### Mice and murine tumor studies

All animal procedures reported in this study were approved by the NCI Animal Care and Use Committee (ACUC) and in accordance with federal regulatory requirements and standards. All components of the intramural NIH ACU program are accredited by Association for Assessment and Accreditation of Laboratory Animal Care (AAALAC) International. In all animal experiments with subcutaneous (s.c.) flank tumors, tumors did not exceed 20 mm in any dimension, the maximum size specified in the approved animal protocol. Mice bearing orthotopic HNSCC tumors were monitored closely for weight loss which did not exceed a decrease of 15 percent body weight.

NSG-MHC I/II KO mice (NOD.Cg-*Prkdc*^*scid*^* H2-K1*^*tm1Bpe*^* H2-Ab1*^*em1Mvw*^* H2-D1*^*tm1Bpe*^* Il2rg*^*tm1Wjl*^/SzJ) were obtained from The Jackson Laboratory and bred in house. UM-SCC-74B cells (1 × 10^6^) or UM-SCC-11B cells (1 × 10^6^) were implanted s.c. in a 3:1 suspension of PBS and Matrigel (Corning), and UPCI-SCC-90 cells (3 × 10^6^) were implanted s.c. in a 1:1 suspension of PBS and Matrigel in the flank of female NSG-MHC I/II KO mice (day 0). C57BL/6 mice were obtained from the NCI Frederick Cancer Research Facility. MOC1 cells (4 × 10^6^) were implanted s.c. in a 3:1 suspension of PBS and Matrigel in the flank of C57BL/6 mice (day 0). In some studies, MOC1 cells (1 × 10^6^) were implanted orthotopically into the tongues of anesthetized C57BL/6 mice in a 1:1 suspension of PBS and Matrigel in a volume of 20 µl. Mice were randomized based on tumor size at the start of drug treatment to equalize the average tumor volume in each group. Control diet feed or SX-682-containing feed (200 mg/kg body weight/day; Dyets, Bethlehem, PA) was administered to mice starting on day 6 or 7. In some experiments, docetaxel (5 mg/kg) was administered as indicated with the first dose being concurrent with the start of SX-682 or control feed. At experimental endpoint, mice were euthanized, and tumors were collected and fixed in Z-Fix (Anatech LTC, Battle Creek, MI), stored in RNAlater (Invitrogen), or dissociated for analysis. In all experiments, tumors were measured with a vernier caliper in two perpendicular diameters. Tumor volume = (short diameter^2^ x long diameter)/2.

### Depletion studies

To deplete immune cells from MOC1 tumor-bearing mice, 100 μg of anti-CD8 (clone 2.43, catalog BP0061, BioXcell, Lebanon, NH) was administered intraperitoneally (i.p.) on days 5, 7, and 11 after tumor implantation and then once per week; 200 µg of anti-Ly6G (clone 1A8, catalog BE0075-1, BioXcell) was administered i.p. every other day starting on day 5 for the duration of the experiment. In this study, mice were randomized as described above with SX-682-containing feed administered starting on day 5 and docetaxel (5 mg/kg) administered twice per week starting on day 5.

### Flow cytometry

Tumors were weighed, mechanically dissociated with surgical scissors, enzymatically dissociated with 5 mg/mL collagenases I and IV (Gibco) and 40 U/mL DNase in media in a 37 °C shaker and passed through a 70 µM filter to form a single cell suspension. Cells were stained in round-bottom 96-well plates on ice in PBS with 2% FBS, and intracellular markers were stained using the eBioscience Foxp3/Transcription Factor Staining Buffer Set according to the manufacturer’s instructions. All fluorescently conjugated antibodies, including CD11b (clone M1/70, Biolegend, San Diego, CA), Ly6G (clone 1A8, Biolegend), CD3e (clone 500A2, Biolegend), CD8 (clone 53–6.7, Biolegend), and granzyme B (clone QA18A28, Biolegend), were used per the manufacturers’ instructions. LIVE/DEAD Fixable Aqua Dead Cell Stain Kit (ThermoFisher Scientific) was used to gate on live cells. Data were acquired on an Attune NxT Flow Cytometer (ThermoFisher Scientific) and analyzed via FlowJo (Becton Dickinson, Washington, DC).

### Statistical analysis methods

Data were graphed and analyzed using GraphPad Prism. Significance was determined by a two-tailed, unpaired Student’s t-test for data sets consisting of two groups or a one-way ANOVA with Tukey’s post hoc test for three or more sets of data. Tumor and cell growth curve analysis was conducted using a two-way ANOVA. Data points in graphs represent mean ± SD or SEM where indicated. *, *P* < 0.05; **, *P* < 0.01; ***,* P* < 0.001; ****,P < 0.0001.

## Results

### Expression of IL-8 and CXCR1/2 in HNSCC tumors

**Fig. 1:**
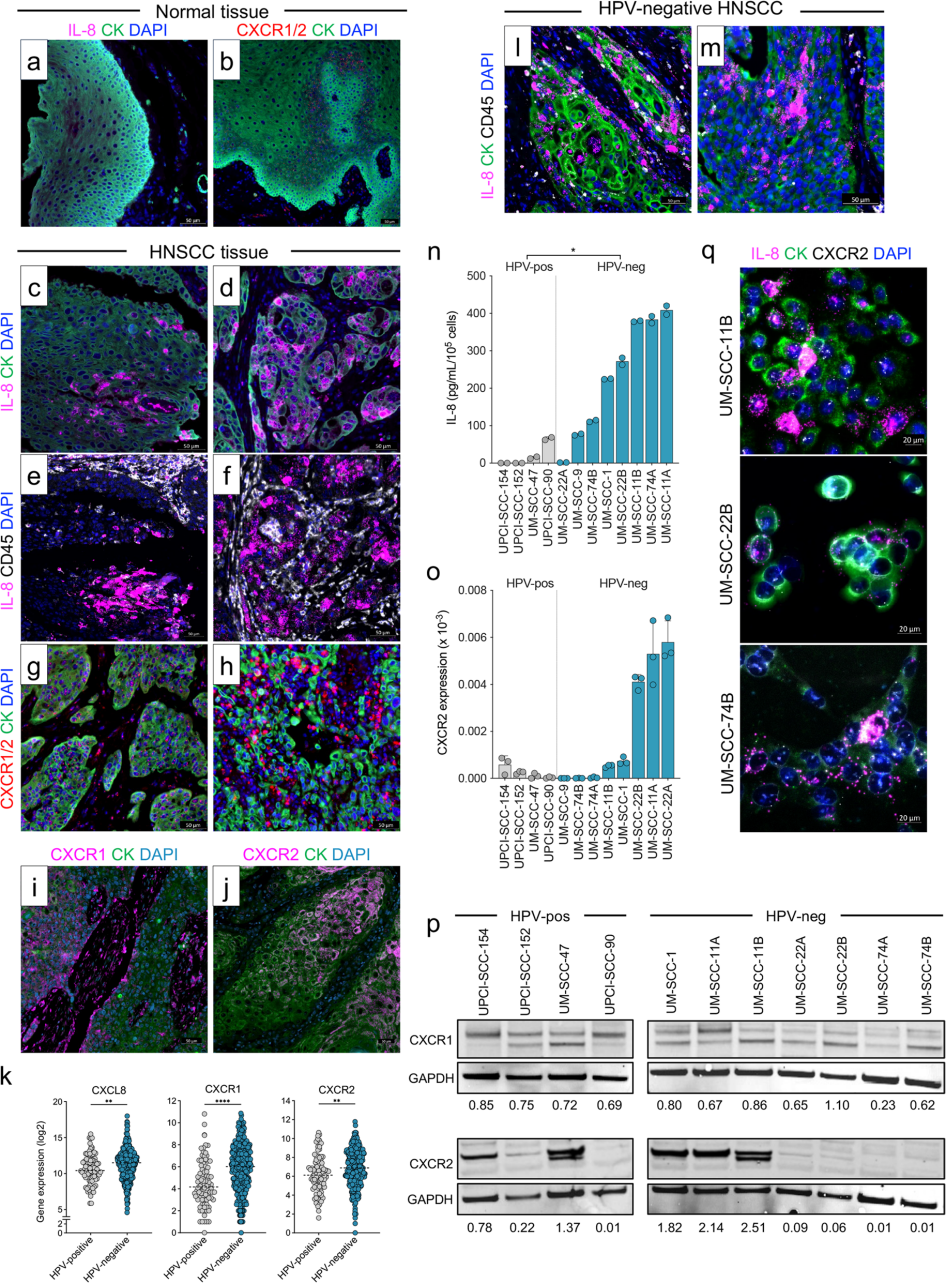
Expression of IL-8 and CXCR1/2 in HNSCC. **a**-**h** Representative images of tissues from a head and neck cancer tumor tissue array stained for IL-8 mRNA (pink), pan-cytokeratin (CK, green), CXCR1 and CXCR2 mRNA (CXCR1/2, red), or CD45 (white). **a**, **b** Representative cases corresponding to normal pharyngeal mucosa (**a**) and normal tongue (**b**). **c**-**h** Representative cases of squamous cell carcinoma of larynx stage IVA (**c**, **e**), epiglottis stage III (**d**, **f**), two cases of squamous cell carcinoma of larynx stage III (**g**, **h**). **i** A case of squamous cell carcinoma of the laryngopharynx stage III stained with antibodies against CXCR1 (pink) and CK (green). **j** A case of nasopharynx stage I stained with antibodies against CXCR2 (pink) and CK (green). **k** RNAseq data from the TCGA head and neck cancer database analyzed for expression of CXCL8, CXCR1, and CXCR2 in HPV-negative (*n* = 410) and HPV-positive (*n* = 90) tumors. **l**, **m** Representative images of HPV-negative tumor tissues from a head and neck cancer tumor tissue array stained for IL-8 mRNA (pink), pan-cytokeratin (CK, green), and CD45 (white) corresponding to squamous cell carcinoma of pharynx stage I (**l**) and pharynx stage II (**m**). **n** IL-8 secreted by HPV-negative and HPV-positive HNSCC cell lines in 48-h culture supernatants determined by ELISA and normalized to cell counts at time of collection. **o** RT-PCR analysis of CXCR2 gene expression in HNSCC cell lines, separated by HPV status. **p** Expression of CXCR1 and CXCR2 proteins in HPV-positive and HPV-negative HNSCC cell lines via immunoblot analysis. GAPDH was detected on the same membranes as a loading control. Numbers below blots indicate expression of the protein of interest as a ratio compared to the corresponding GAPDH. **q** Representative images of HPV-negative HNSCC cell lines stained for IL-8 mRNA (pink), pan-cytokeratin (CK, green), and CXCR2 mRNA (white). DAPI (blue) was used to stain nuclei in all representative images. Values graphed in (**n**, **o**) represent the mean ± SEM of technical replicates (*n* = 2 for ELISA assay; *n* = 3 for RT-PCR). Results are representative of 2 independent experiments. * *p* < 0.05, ** *p* < 0.01, **** *p* < 0.0001 for Student’s t-test in (**k**, **n**)

Expression and localization of IL-8, CXCR1, and CXCR2 in normal tissues (Fig. 1 a-b) and HNSCC tumors (Fig. 1 c-h) were evaluated with a commercially available tissue array containing 70 tumor cases of unknown HPV status and 10 normal tissues. Tissues were co-stained for expression of IL-8 or CXCR1/2 mRNA via RNA in situ hybridization and either pan-cytokeratin (CK) or CD45 via immunofluorescence. While IL-8 mRNA was undetectable in normal tissues (Fig. 1a and Table 1), IL-8 mRNA was either co-expressed with CK indicating direct expression in cancer cells (scored as CK^POS^IL-8^POS^ in Table 1; 42% of cases) or detected in CK negative cells (scored as CK^NEG^IL-8^POS^ in Table 1; 46.4% of cases) in the HNSCC tissues evaluated (Fig. 1c-d and Table 1). In general, a pattern of expression corresponding to clusters of IL-8 positive cells was observed across tissues (Fig. 1c-d). To understand whether IL-8 detected in CK negative cells was localized to immune cells in the TME, tissues were co-stained for the immune cell marker CD45 and IL-8 mRNA. As shown with representative images in Fig. 1e-f, IL-8 mRNA was detected in only a fraction of CD45^+^ infiltrating immune cells, while most of the expression was localized to non-immune cells. RNA in situ hybridization was also employed to evaluate expression of the IL-8 receptors using a combination of probes directed against CXCR1 and CXCR2 (Fig. 1b, g-h, and Table 1). CXCR1/2 mRNA was found to be expressed in normal epithelial cells (Fig. 1b), CK positive tumor cells (Fig. 1g), and CK negative cells in the TME (Fig. 1h). Overall, 76/80 HNSCC tissues analyzed expressed CXCR1/2 mRNA (Table 1). The protein expression of both CXCR1 and CXCR2 was further confirmed through multiplex immunofluorescence analysis employing an additional commercially available tissue array containing 78 HNSCC cases (Supp. Table 1). Overall, CXCR1 protein was detected in tumor CK positive and stromal CK negative cells (Fig. 1i and Supp. Table 1) while CXCR2 protein was predominantly detected in CK positive tumor cells (Fig. 1j and Supp. Table 1). Altogether, the data indicated that HNSCC tumors overexpress IL-8 and are positive for the expression of CXCR1 and CXCR2, potentially making this pathway an actionable target in the context of HNSCC.

**Table 1:** Analysis of IL-8, CXCR1, and CXCR2 mRNA expression via RNA in situ hybridization in a head and neck cancer tissue array

**No.**	**Organ**	**Pathology Diagnosis**	** Grade**	**Stage**	**TNM**	**Type**	**CK**^**POS**^ **IL-8**^**POS**^	**CK**^**NEG**^ **IL-8**^**POS**^	**CK**^**POS**^ **CXCR1/2**^**POS**^	**CK**^**NEG**^ **CXCR1/2**^**POS**^
1	Larynx	Larynx tissue	-	-	-	Normal	-	-	*Tissue lost*	
2	Pharynx	Pharyngeal mucosa tissue	-	-	-	Normal	-	-	Pos	-
3	Pharynx	Pharyngeal mucosa tissue	-	-	-	Normal	-	-	-	Pos
4	Pharynx	Pharyngeal mucosa tissue	-	-	-	Normal	-	-	*Tissue lost*	
5	Pharynx	Pharyngeal mucosa tissue	-	-	-	Normal	-	-	Pos	-
6	Tongue	Tongue tissue	-	-	-	Normal	-	-	Pos	-
7	Tongue	Tongue tissue	-	-	-	Normal	-	-	Pos	Pos
8	Tongue	Tongue tissue	-	-	-	Normal	-	-	Pos	-
9	Tongue	Tongue tissue	-	-	-	Normal	-	-	Pos	-
10	Tongue	Tongue tissue	-	-	-	Normal	-	-	Pos	-
11	Nose	Non keratinizing squamous cell ca.	3	II	T2N0M0	Malignant	-	-	Pos	Pos
12	Larynx	Non keratinizing squamous cell ca.	2-3	II	T2N0M0	Malignant	Pos	-	Pos	Pos
13	Larynx	Non keratinizing squamous cell ca.	3	III	T3N0M0	Malignant	Pos	Pos	Pos	Pos
14	Larynx	Non keratinizing squamous cell ca.	2-3	III	T3N1M0	Malignant	-	Pos	Pos	Pos
15	LN	Non keratinizing squamous cell ca.	3	II	T2N0M0	Malignant	Pos	Pos	Pos	Pos
16	Larynx	Non keratinizing squamous cell ca.	3	I	T1N0M0	Malignant	-	-	Pos	-
17	Pharynx	Non keratinizing squamous cell ca.	3	III	T3N0M0	Malignant	-	-	Pos	Pos
18	Nose	Non keratinizing squamous cell ca.	3	I	T1N0M0	Malignant	Pos	Pos	Pos	Pos
19	Larynx	Non keratinizing squamous cell ca.	3	III	T3N0M0	Malignant	-	-	-	-
20	Larynx	Non keratinizing squamous cell ca.	2-3	II	T2N0M0	Malignant	Pos	Pos	-	Pos
21	Larynx	Non keratinizing squamous cell ca.	2	III	T3N0M0	Malignant	-	-	Pos	Pos
22	Larynx	Non keratinizing squamous cell ca.	2	IVA	T4aN0M0	Malignant	-	-	Pos	Pos
23	Larynx	Non keratinizing squamous cell ca.	3	IVA	T1N2bM0	Malignant	Pos	-	-	Pos
24	Larynx	Keratinizing squamous cell ca.	2	III	T3N0M0	Malignant	Pos	-	Pos	Pos
25	Larynx	Non keratinizing squamous cell ca.	3	II	T2N0M0	Malignant	Pos	Pos	Pos	Pos
26	Nose	Non keratinizing squamous cell ca.	2	I	T1N0M0	Malignant	-	Pos	-	-
27	Larynx	Keratinizing squamous cell ca.	3	II	T2N0M0	Malignant	-	-	-	Pos
28	Larynx	Keratinizing squamous cell ca.	2	III	T2N1M0	Malignant	-	-	Pos	Pos
29	Larynx	Keratinizing squamous cell ca.	2-3	IVA	T3N2bM0	Malignant	Pos	-	Pos	Pos
30	Larynx	Keratinizing squamous cell ca.	3	IVA	T2N2aM0	Malignant	-	-	Pos	Pos
31	Larynx	Keratinizing squamous cell ca.	2-3	IVA	T3N2bM0	Malignant	-	-	-	Pos
32	Larynx	Keratinizing squamous cell ca.	2	II	T2N0M0	Malignant	-	-	-	-
33	Larynx	Keratinizing squamous cell ca.	2	I	T1N0M0	Malignant	-	-	Pos	-
34	Larynx	Keratinizing squamous cell ca.	2	IVA	T4aN0M0	Malignant	Pos	Pos	Pos	Pos
35	Nose	Non keratinizing squamous cell ca.	3	I	T1N0M0	Malignant	-	-	-	Pos
36	Larynx	Non keratinizing squamous cell ca.	3	III	T3N0M0	Malignant	Pos	Pos	Pos	Pos
37	Pharynx	Keratinizing squamous cell ca.	2	III	T3N0M0	Malignant	-	-	Pos	-
38	Tongue	Keratinizing squamous cell ca.	2	III	T3N0M0	Malignant	Pos	-	Pos	Pos
39	Epiglottis	Keratinizing squamous cell ca.	2	III	T3N0M0	Malignant	-	Pos	Pos	Pos
40	Larynx	Keratinizing squamous cell ca.	2	IVA	T3N2bM0	Malignant	-	-	Pos	Pos
41	Gums	Keratinizing squamous cell ca.	2	II	T2N0M0	Malignant	Pos	Pos	*Tissue lost*	
42	Larynx	Keratinizing squamous cell ca.	2	II	T2N0M0	Malignant	Pos	-	Pos	Pos
43	Larynx	Keratinizing squamous cell ca.	*	IVA	T4aN0M0	Malignant	-	-	*Tissue lost*	
44	Pharynx	Keratinizing squamous cell ca.	1-2	II	T2N0M0	Malignant	-	Pos	-	Pos
45	Larynx	Keratinizing squamous cell ca.	2-3	II	T2N0M0	Malignant	-	-	-	Pos
46	Gums	Non keratinizing squamous cell ca.	1	III	T3N0M0	Malignant	-	-	Pos	Pos
47	Larynx	Non keratinizing squamous cell ca.	1	II	T2N0M0	Malignant	Pos	Pos	Pos	Pos
48	Epiglottis	Keratinizing squamous cell ca.	2	III	T2N1M0	Malignant	Pos	-	Pos	Pos
49	Larynx	Keratinizing squamous cell ca.	2	IVA	T4aN0M0	Malignant	Pos	-	Pos	Pos
50	Larynx	Keratinizing squamous cell ca.	1	IVA	T4aN1M0	Malignant	-	Pos	Pos	Pos
51	Larynx	Keratinizing squamous cell ca.	2	IVA	T4aN2cM0	Malignant	-	Pos	Pos	Pos
52	Larynx	Keratinizing squamous cell ca.	2	IVA	T3N2cM0	Malignant	*Tissue lost*		*Tissue lost*	
53	Larynx	Keratinizing squamous cell ca.	2	III	T3N0M0	Malignant	Pos	-	Pos	Pos
54	Larynx	Keratinizing squamous cell ca.	2-3	III	T3N1M0	Malignant	Pos	-	Pos	-
55	Larynx	Keratinizing squamous cell ca.	1	III	T3N0M0	Malignant	-	-	-	Pos
56	Oral Cav.	Keratinizing squamous cell ca.	1	I	T2N0M0	Malignant	-	-	-	Pos
57	Tongue	Keratinizing squamous cell ca.	1	I	T1N0M0	Malignant	-	Pos	Pos	Pos
58	Larynx	Keratinizing squamous cell ca.	2	IVA	T3N2cM0	Malignant	-	Pos	*Tissue lost*	
59	Tongue	Keratinizing squamous cell ca.	1	IVA	T2N2bM0	Malignant	-	Pos	*Tissue lost*	
60	Tongue	Keratinizing squamous cell ca.	1	II	T2N0M0	Malignant	Pos	Pos	Pos	Pos
61	Larynx	Keratinizing squamous cell ca.	1	IVA	T4aN0M0	Malignant	-	-	Pos	Pos
62	Larynx	Keratinizing squamous cell ca.	1	IVA	T4aN0M0	Malignant	-	Pos	Pos	Pos
63	Larynx	Keratinizing squamous cell ca.	1	I	T1N0M0	Malignant	Pos	Pos	Pos	Pos
64	Larynx	Keratinizing squamous cell ca.	1	IVA	T4aN0M0	Malignant	-	-	Pos	Pos
65	Larynx	Keratinizing squamous cell ca.	1	III	T3N0M0	Malignant	-	-	Pos	Pos
66	Larynx	Keratinizing squamous cell ca.	1	IVA	T4aN0M0	Malignant	Pos	Pos	Pos	Pos
67	Larynx	Keratinizing squamous cell ca.	1	IVA	T4aN2bM0	Malignant	Pos	Pos	Pos	Pos
68	Epiglottis	Keratinizing squamous cell ca.	1	IVA	T4aN0M0	Malignant	-	-	-	-
69	Tongue	Keratinizing squamous cell ca.	1	III	T3N0M0	Malignant	-	Pos	*Tissue lost*	
70	Larynx	Keratinizing squamous cell ca.	1	IVA	T2N2bM0	Malignant	-	Pos	*Tissue lost*	
71	Larynx	Keratinizing squamous cell ca.	1	IVA	T4aN0M0	Malignant	-	-	Pos	Pos
72	Larynx	Keratinizing squamous cell ca.	1	IVA	T4aN0M0	Malignant	-	-	Pos	Pos
73	Lip	Keratinizing squamous cell ca.	1	II	T2N0M0	Malignant	Pos	Pos	Pos	Pos
74	Tongue	Keratinizing squamous cell ca.	1	II	T2N0M0	Malignant	Pos	Pos	Pos	Pos
75	Larynx	Keratinizing squamous cell ca.	1	III	T3N0M0	Malignant	-	-	-	Pos
76	Larynx	Keratinizing squamous cell ca.	1	III	T3N0M0	Malignant	Pos	Pos	Pos	Pos
77	Tongue	Keratinizing squamous cell ca.	1	I	T1N0M0	Malignant	Pos	Pos	Pos	Pos
78	Soft pal.	Keratinizing squamous cell ca.	1	IVA	T3N2bM0	Malignant	Pos	Pos	Pos	Pos
79	Larynx	Keratinizing squamous cell ca.	1	II	T2N0M0	Malignant	-	Pos	-	Pos
80	Larynx	Keratinizing squamous cell ca.	1	II	T2N0M0	Malignant	Pos	Pos	Pos	Pos
**Normal tissues % positive cases**							**0.0%**	**0.0% **	**87.5%**	**25.0%**
**Tumor tissues % positive cases**							**42.0%**	**46.4%**	**76.2%**	**87.3%**

*LN* lymph node, *Oral Cav* oral cavity, *Soft pal* soft palate, *Pos* positive, (-) negative

### Predominant expression of IL-8 and CXCR1/2 in HPV-negative HNSCC

To investigate whether IL-8 and its receptors are equally expressed across HPV-negative and HPV-positive HNSCC tumors, RNAseq data in the TCGA head and neck tumor database were analyzed. Expression of IL-8 was significantly higher in HPV-negative compared to HPV-positive primary HNSCC tumors (Fig. 1k). This observation was confirmed through multiplex immunofluorescence staining of a commercially available tissue array that included p16 scoring as a surrogate for HPV status (Fig. 1l-m and Table 2). Tissues were co-stained for expression of IL-8 via RNA in situ hybridization and CK and CD45 via immunofluorescence. Overall, a higher frequency of HPV-negative tissues expressed IL-8 mRNA in both CK positive and CK negative cells (Table 2). TCGA analysis also revealed that CXCR1 and CXCR2 were predominantly expressed in HPV-negative compared to HPV-positive primary HNSCC tumors (Fig. 1k). To explore transcriptomic features associated with IL-8 expression in HNSCC tumors, HPV-negative HNSCC cases from the TCGA database (*n* = 410) were sorted by high to low IL-8 expression and divided into two equal groups (IL-8-High vs. IL-8-Low) for comparison. Differential gene expression analysis revealed that IL-8-High tumors, in addition to higher expression of CXCR1 and CXCR2, expressed higher levels of various genes encoding for other CXCL family chemokines (Supp. Figure 1a) including *CXCL1, CXCL2, CXCL3, CXCL5*, and *CXCL6*. Additionally, genes indicating an inflamed microenvironment were more highly expressed in IL-8-High versus IL-8-Low tumors, including *IL6, CSF2, CSF3, IL1A,* and *IL1B*. Pathway analysis showed activation of TNF-α signaling via NF-kB, inflammatory response, IL-10 signaling, and EMT (Supp. Figure 1b), among others. The prevalence of IL-8, CXCR1, and CXCR2 in HPV-negative versus HPV-positive HNSCC was further demonstrated with a panel of human HNSCC cell lines. As shown in Fig. 1n, 7/8 HPV-negative HNSCC cell lines secreted IL-8 in excess of 100 pg/mL per 10^5^ cells, while none of the HPV-positive lines secreted IL-8 above this level in the culture supernatant. Similarly, expression of CXCR2 mRNA was high in 3/8 HPV-negative HNSCC lines, while all other HPV-negative and HPV-positive cell lines expressed low levels of CXCR2 mRNA (Fig. 1o). CXCR1 and CXCR2 protein expression in HNSCC cell lines quantified by immunoblot (Fig. 1p and Supp. Figure 2) did not reveal differences between HPV-negative and HPV-positive cell lines. Interestingly, an increase in the expression of the CXCR1 receptor was observed in the HPV-negative lines derived from metastatic, post-therapy, and persistent tumors (UM-SCC-11B, −22B, and −74B) compared to lines derived from primary or pre-treatment biopsied tumors (UM-SCC-11A, −22A, and −74A) [22]. Based on these findings and the observations made with tumors in the TCGA database, we focused our subsequent studies on three HPV-negative cell line models, UM-SCC-11B, UM-SCC-22B, and UM-SCC-74B, with various levels of IL-8 secretion. RNA in situ hybridization for IL-8 and CXCR2 mRNA, and CK immunofluorescence (Fig. 1q) on these cell lines further confirmed expression of these targets and highlighted the intriguing heterogeneity of IL-8 expression within each line.

**Table 2:** Analysis of IL-8 mRNA expression via RNA in situ hybridization in a tissue array containing p16 positive and negative head and neck cancer tissues

**No.**	**Organ**	**Grade**	**Stage**	**TNM**	**p16**	**CK**^**POS**^ **IL-8**^**POS**^	**CK**^**NEG**^ **IL-8**^**POS**^
1	Nose	1	I	T1N0M0	-	-	-
2	Nose	2	I	T1N0M0	-	-	-
3	Nose	2	III	T3N0M0	+	-	-
4	Nose	2	I	T1N0M0	-	Pos	Pos
5	Nose	3	III	T3N0M0	-	Pos	Pos
6	Nose	3	II	T2N0M0	-	No Tumor Tissue	
7	Nasopharynx	2	II	T2N0M0	-	-	-
8	Nasopharynx	2	II	T2N0M0	+++	-	-
9	Maxillary sinus	1	I	T1N0M0	-	-	-
10	Maxillary sinus	2	I	T1N0M0	+	-	-
11	Maxillary sinus	2	II	T2N0M0	-	Pos	-
12	Nose	3	II	T2N0M0	-	-	-
13	Maxillary sinus	3	II	T2N0M0	-	Pos	-
14	Maxillary sinus	3	I	T1N0M0	-	-	-
15	Maxillary sinus	3	III	T3N0M0	+++	-	Pos
16	Ethmoid sinus	3	II	T2N0M0	-	Tissue Lost	
17	Pharynx	*	III	T2N1M0	-	No Tumor Tissue	
18	Pharynx	1	III	T3N0M0	-	-	-
19	Pharynx	2	II	T2N0M0	-	-	Pos
20	Pharynx	1	IVA	T4aN0M0	-	Pos	Pos
21	Pharynx	2	II	T2N0M0	-	-	-
22	Pharynx	2	IVA	T4aN0M0	++	-	-
23	Pharynx	1	II	T2N0M0	-	-	Pos
24	Pharynx	1	IVA	T3N2M0	-	-	-
25	Pharynx	1	I	T1N0M0	-	-	-
26	Pharynx	2	IVA	T4aN0M0	-	Pos	Pos
27	Pharynx	2	II	T2N0M0	-	-	-
28	Pharynx	2	II	T2N0M0	-	-	-
29	Pharynx	2	III	T2N1M0	+++	-	-
30	Pharynx	2	III	T3N0M0	-	Pos	Pos
31	Pharynx	2	II	T2N0M0	-	Pos	Pos
32	Pharynx	2	III	T3N0M0	-	-	-
33	Pharynx	2	III	T2N1M0	+++	Pos	Pos
34	Pharynx	3	II	T2N0M0	-	-	-
35	Laryngopharynx	1	II	T2N0M0	-	Pos	-
36	Laryngopharynx	1	I	T1N0M0	++	-	-
37	Laryngopharynx	1	II	T2N0M0	++	Pos	-
38	Laryngopharynx	1	II	T2N0M0	-	-	-
39	Laryngopharynx	1	II	T2N0M0	-	-	-
40	Laryngopharynx	1	I	T1N0M0	-	Pos	Pos
41	Laryngopharynx	2	IVA	T4aN0M0	-	Pos	-
42	Laryngopharynx	2	III	T2N1M0	-	Pos	-
43	Laryngopharynx	2	III	T2N1M0	-	-	-
44	Laryngopharynx	2	II	T2N0M0	-	-	-
45	Laryngopharynx	1	II	T2N0M0	-	-	-
46	Laryngopharynx	2	II	T2N0M0	-	-	-
47	Laryngopharynx	2	II	T2N0M0	-	-	Pos
48	Laryngopharynx	2	II	T2N0M0	-	Pos	Pos
49	Laryngopharynx	2	II	T2N0M0	++	-	-
50	Laryngopharynx	2	III	T2N1M0	-	-	-
51	Laryngopharynx	2	IVA	T2N2M0	-	-	-
52	Laryngopharynx	2	II	T2N0M0	-	Pos	Pos
53	Laryngopharynx	2	IVA	T4aN0M0	-	-	-
54	Laryngopharynx	3	II	T2N0M0	-	-	-
55	Laryngopharynx	2	III	T2N1M0	-	-	Pos
56	Laryngopharynx	3	IVA	T4aN1M0	-	Pos	-
57	Larynx	1	II	T2N0M0	-	-	-
58	Larynx	1	III	T2N1M0	-	-	-
59	Larynx	1	IVA	T4aN0M0	++	-	-
60	Larynx	2	III	T2N1M0	-	-	Pos
**Percent positive (p16 positive cases)**						**18.2% **	**18.2%**
**Percent positive (p16 negative cases)**						**32.6% **	**30.4%**

*Pos* positive, (-) negative

### Blockade of CXCR1/2 selectively sensitizes HPV-negative HNSCC cells to docetaxel

**Fig. 2:**
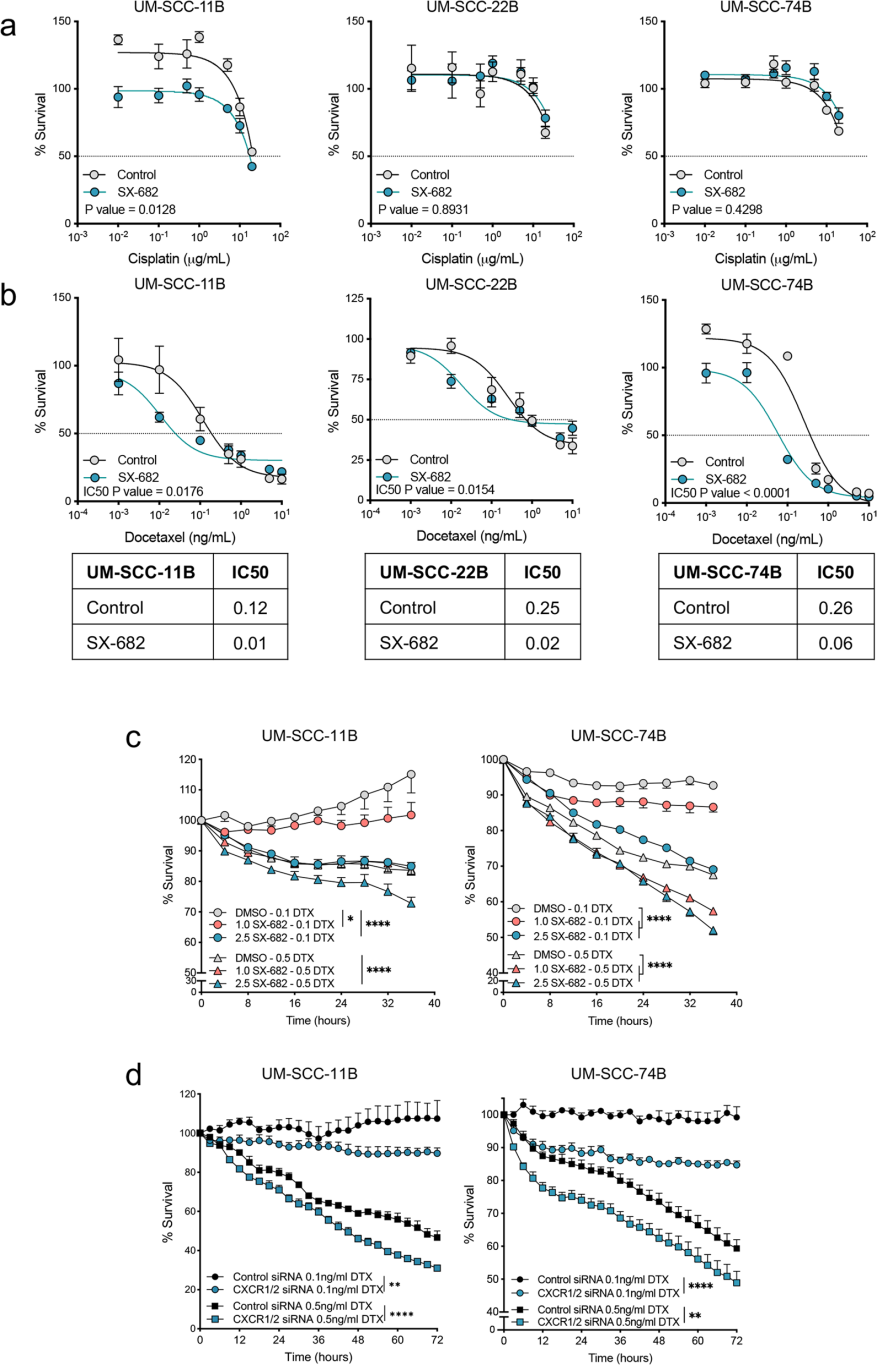
Inhibition of the CXCR1/2 pathway improves susceptibility of HPV-negative HNSCC to docetaxel in vitro. **a**, **b** Indicated cell lines were pre-treated with DMSO (Control) or SX-682 (10 µM) for 72 h, followed by treatment with DMSO or SX-682 and a range of cisplatin (**a**) or docetaxel (**b**) concentrations for 72 h. A CellTiterGlo assay was used to determine % survival of cells and GraphPad Prism was used to calculate an IC50 value. Values represent the mean ± SEM of technical replicates and the solid line is a calculated nonlinear regression. Dotted horizontal line plotted at 50% survival. **c** Proliferation of indicated cell lines pre-treated with DMSO, 1 µM SX-682 (orange), or 2.5 µM SX-682 (teal) and treated with either 0.1 ng/mL docetaxel (circles) or 0.5 ng/mL docetaxel (triangles). **d** Proliferation of indicated cell lines transfected with control siRNA (black) or a pool of CXCR1/2 siRNA (teal) and treated with either 0.1 ng/mL docetaxel (circles) or 0.5 ng/mL docetaxel (triangles). Values represent the mean ± SEM of technical replicates. Results in **a**, **b**, **c**, and **d** are representative of 2 independent experiments. IC50 values were compared using an extra sum-of-squares F test in (**a**, **b**). * *p* < 0.05, ** *p* < 0.01, **** *p* < 0.0001 for 2-way ANOVA in (**c**, **d**)

Given that IL-8 has been implicated in tumor resistance to a broad number of cancer therapies, we evaluated whether blockade of IL-8 signaling via inhibition of the CXCR1/2 receptors may improve the response of HPV-negative HNSCC to various chemotherapies. Inhibition of CXCR1/2 was achieved via treatment of tumor cells in culture with the small molecule inhibitor, SX-682; chemotherapy agents included the alkylating agent cisplatin and the microtubule-targeting agent, docetaxel. Titration of cisplatin and docetaxel responses with all three HPV-negative HNSCC cell lines showed no effect of 10 µM SX-682 pretreatment on cisplatin sensitivity (Fig. 2a) while consistently lowering IC50 values for docetaxel (Fig. 2b). The docetaxel IC50 values for untreated and SX-682 treated cells, respectively, were 0.12 and 0.01 ng/mL for UM-SCC-11B, 0.25 and 0.02 ng/mL for UM-SCC-22B, and 0.26 and 0.06 ng/mL for UM-SCC-74B cells (Fig. 2b). To test the ability of SX-682 to sensitize tumor cells to docetaxel at lower drug concentrations, UM-SCC-11B and UM-SCC-74B cells were pretreated with SX-682 at either 1 µM or 2.5 µM and evaluated in a proliferation assay with low (0.1 ng/mL) or high (0.5 ng/mL) treatment doses of docetaxel (Fig. 2c). Both cell lines were modestly sensitized to docetaxel with 1 µM SX-682 pre-treatment while pre-treatment with 2.5 µM SX-682 resulted in a significant decrease in cell proliferation in response to docetaxel treatment. Finally, the use of UM-SCC-11B and UM-SCC-74B cells transfected with siRNA targeting CXCR1 and CXCR2 (Fig. 2d) further confirmed that removal of CXCR1/2 receptor signaling could sensitize HPV-negative HNSCC tumor cells to the cytotoxic activity of docetaxel in vitro.

### Inhibition of CXCR1/2 signaling modulates tumor cell phenotype to increase susceptibility to docetaxel

**Fig. 3:**
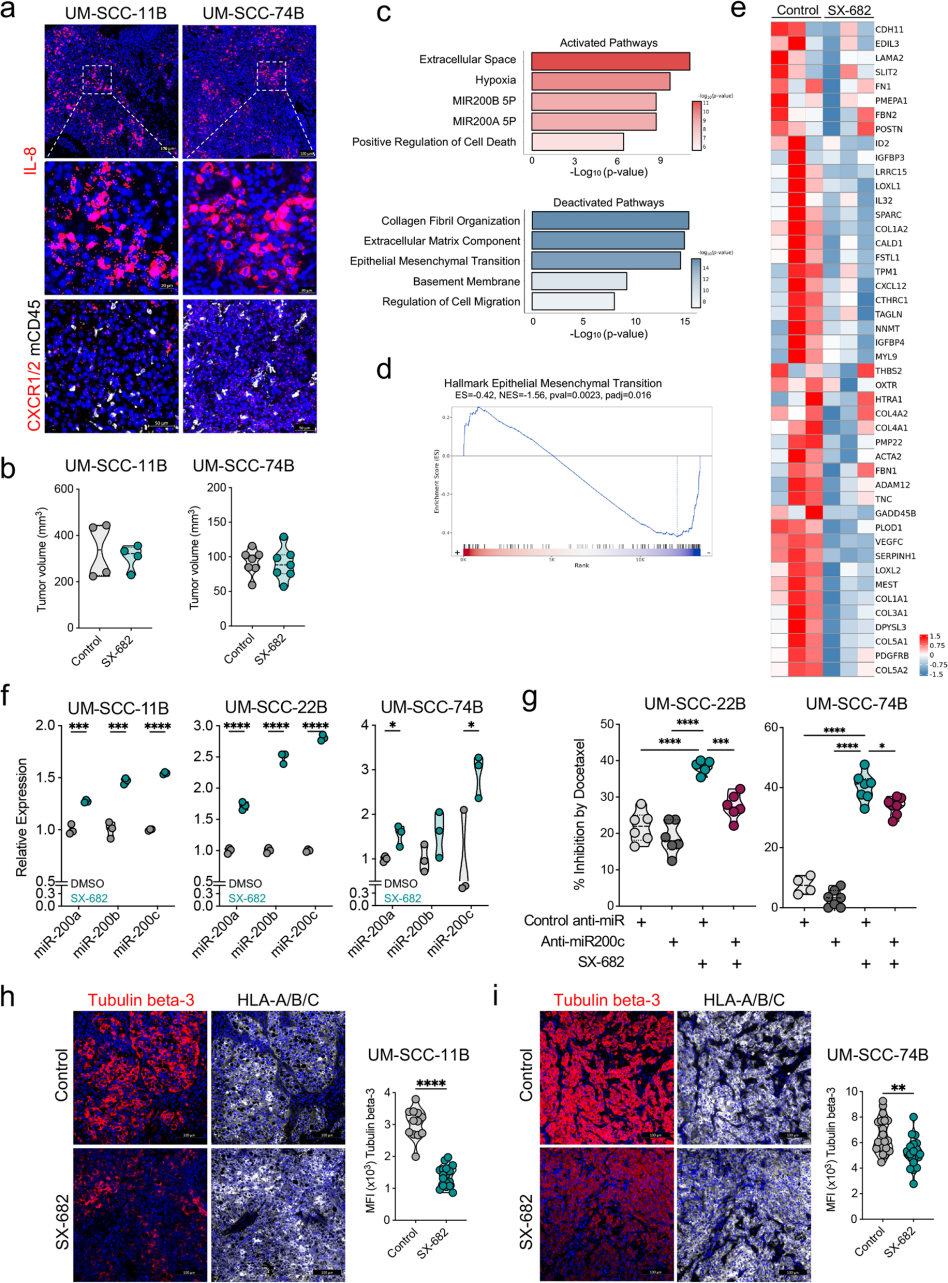
SX-682 modulates the phenotype of HPV-negative HNSCC xenografts and upregulates miR-200. **a** UM-SCC-11B and UM-SCC-74B xenografts grown in NSG-MHC I/II knockout mice were evaluated via RNA in situ hybridization for expression of IL-8 or CXCR1/2 mRNA (red) and staining for CD45 (white). **b** Final tumor volumes of UM-SCC-11B and UM-SCC-74B xenografts on day 15 post-tumor implantation in mice receiving a control or a SX-682-containing feed starting on day 7 or day 6, respectively. **c** RNAseq analysis on control and SX-682 treated UM-SCC-74B xenografts. Shown are selected activated and deactivated GO, HALLMARK, and C3 pathways when comparing SX-682 to control. **d** GSEA Enrichment plot showing normalized enrichment score by ranked genes for HALLMARK Epithelial-Mesenchymal Transition pathway. **e** Heatmap of leading-edge genes identified in (**d**). Data represents *n* = 3 mice per group. **f** Cell lines treated with SX-682 (teal) or DMSO control (gray) for 24–48 h, followed by analysis of miR-200a, miR-200b, and miR-200c by RT-PCR. Expression was normalized to expression of snoRNA135 and graphed as relative to DMSO cells. **g** UM-SCC-22B and UM-SCC-74B cells were pre-treated with SX-682 and transfected with anti-miR200c or a control anti-miR. Cells were subsequently treated with docetaxel (0.75 ng/mL and 0.5 ng/mL respectively) with or without SX-682. Growth inhibition was determined after 72 or 48 h respectively. **h**-**i** Tumors from control and SX-682 treated UM-SCC-11B (**h**) and UM-SCC-74B xenografts (**i**) from (**b**) were collected at endpoint and stained for expression of tubulin beta-3 (red), HLA-A/B/C (white), and DAPI (blue) (*n* = 4 tumors per group). Quantification of the MFI of tubulin beta-3 expression within the regions of HLA-A/B/C expression is also shown; each dot represents an individual region of interest (ROI). Results in **f** and **g** are representative of 2 independent experiments. * *p* < 0.05, ** *p* < 0.01, *** *p* < 0.001, **** *p* < 0.0001 for Student’s t-test in (**f**, **h**, **i**) and for 1-way ANOVA followed by Tukey’s post hoc test in (**g**)

To determine the effect of SX-682 treatment on HPV-negative HNSCC tumors in vivo, xenografts of UM-SCC-11B and UM-SCC-74B tumor cells growing in NSG-MHC I/II KO mice were first evaluated for the expression of IL-8, CXCR1, and CXCR2 via RNA in situ hybridization. As shown in Fig. 3a, clusters of tumor cells positive for IL-8 mRNA were observed, while the expression of CXCR1/2 mRNA was homogeneously distributed across CD45 negative cells in the tumor. Administration of SX-682 in mouse feed (200 mg/kg body weight) had no significant effect on tumor growth as a monotherapy in either model (Fig. 3b). To determine the potential effect of CXCR1/2 inhibition on the phenotype of tumor cells in vivo, bulk RNA sequencing was performed on control and SX-682 treated UM-SCC-74B xenografts. Pathway analysis from differentially expressed genes in the SX-682 treated vs control groups (Fig. 3c) revealed activation of extracellular space, hypoxia, and cell death-associated pathways, while pathways including collagen organization, extracellular matrix organization, and EMT were deactivated (Fig. 3d-e). Interestingly, microRNA (miR)−200 pathways (MIR200A and MIR200B) were significantly upregulated due to SX-682 treatment in vivo (Fig. 3c). Based on previous reports describing a role for miR-200c in sensitizing tumor cells to microtubule-targeting chemotherapies [23, 24], miR-200 family members were further investigated in HPV-negative cell lines treated with SX-682 in culture. Forty-eight-hour treatment with SX-682 significantly increased the expression of miR-200a and miR-200c in all three cell lines while miR-200b was increased in two of the three tumor cell lines evaluated (Fig. 3f). These results suggested that SX-682-mediated miR-200 modulation could be responsible for the improved response to docetaxel. To test this hypothesis, UM-SCC-22B and UM-SCC-74B cells were left untreated or pre-treated with SX-682 overnight and subsequently transfected with a miR-200c specific inhibitor (anti-miR-200c) or a miRNA negative control, followed by exposure to docetaxel. Our results demonstrated that forced down-regulation of miR-200c precluded SX-682-mediated sensitization to docetaxel (Fig. 3g). Differential expression patterns of specific β-tubulin isoforms have been associated with resistance to chemotherapies in various types of cancer [25]. In particular, the expression of tubulin beta-3 has been associated with resistance to microtubule-targeting chemotherapies in several cancer types [26, 27]. As miR-200c can regulate tubulin beta-3 levels [23, 24], SX-682 treated UM-SCC-11B (Fig. 3h) and UM-SCC-74B (Fig. 3i) xenografts were stained for expression of human HLA-A/B/C to identify tumor cells and tubulin beta-3. With both models, expression of tubulin beta-3 was significantly downregulated in tumor cells in xenografts treated with SX-682 compared to control.

## SX-682 increases susceptibility to docetaxel in xenograft HNSCC models

**Fig. 4:**
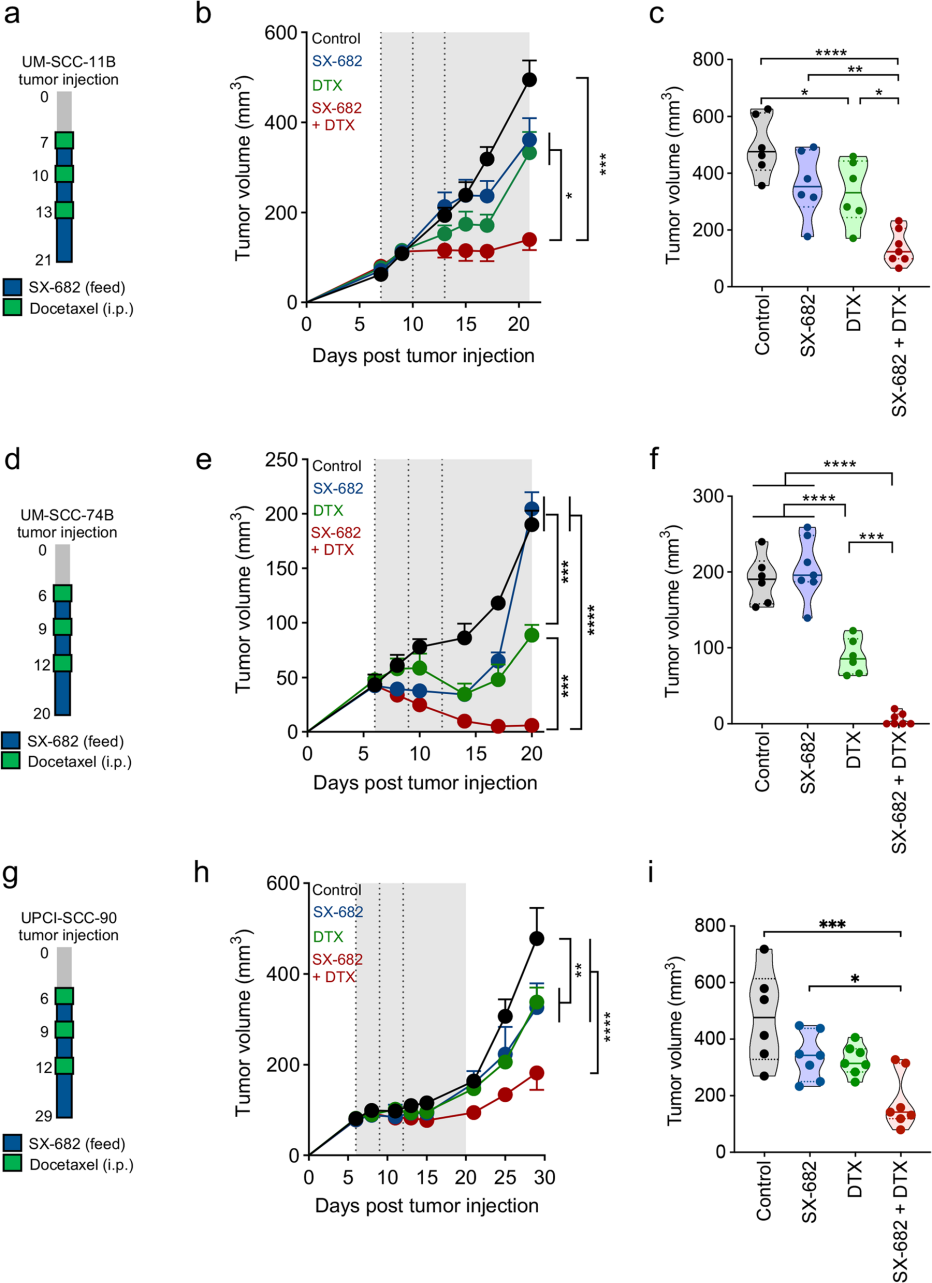
CXCR1/2 inhibition increases susceptibility of HNSCC xenografts to docetaxel in vivo. **a**, **d**, **g** Schematics depicting the timeline of therapy with the UM-SCC-11B, UM-SCC-74B, and UPCI-SCC-90 models, respectively. Control diet or SX-682 feed (200 mg/kg) was administered to mice starting on day 6 or 7, as indicated, for the duration of the experiment. Docetaxel injections (5 mg/kg) were delivered by intraperitoneal (i.p.) injection on indicated days. Average tumor growth and tumor volumes at the end of study, respectively, for the UM-SCC-11B (**b**, **c**), UM-SCC-74B (**e**, **f**), and UPCI-SCC-90 (**h**, **i**) models (*n* = 6–7 mice/group in all studies). Gray shaded area marks the administration of SX-682 while vertical dotted lines denote docetaxel injections. UM-SCC-11B and UM-SCC-74B data are representative of 1 of 2 independent experiments. Values graphed in (**b**, **e**, **h**) represent the mean ± SEM; * *p* < 0.05, ** *p* < 0.01, *** *p* < 0.001, **** *p* < 0.0001 for 2-way ANOVA in (**b**, **e**, **h**), and for 1-way ANOVA followed by Tukey’s post hoc test in (**c**, **f**, **i**)

To determine the effect of SX-682 treatment in combination with docetaxel on HPV-negative HNSCC tumors in vivo, UM-SCC-11B tumors were treated as indicated in Fig. 4a; briefly, mice were fed a control diet or a diet containing SX-682 starting on day 7 and administered docetaxel (5 mg/kg, i.p.) on days 7, 10, and 13. While monotherapy treatment with docetaxel or SX-682 had no significant effect on the growth of this rapidly growing tumor model, the combination of SX-682 and docetaxel exerted robust tumor control compared to the monotherapy treatments or the control group (Fig. 4b-c). The activity of the combination therapy was validated with an additional model. Xenografts of UM-SCC-74B tumor cells were treated as indicated in Fig. 4d. As observed previously, treatment with SX-682 alone had no significant effect on tumor growth; however, docetaxel alone was able to reduce tumor growth without inducing cures. In contrast, a remarkable anti-tumor activity was observed in the combination group resulting in 4/7 (57%) tumor cures in addition to an overall reduction of tumor growth compared to each of the single therapies with SX-682 or docetaxel alone (Fig. 4e-f). The combination of SX-682 plus docetaxel was further tested against the UPCI-SCC-90 HPV-positive HNSCC model characterized by low levels of IL-8 expression in vitro (Fig. 1n). Xenografts of UPCI-SCC-90 tumors treated as indicated (Fig. 4g) only modestly responded to monotherapy treatment with docetaxel or SX-682; however, the combination of SX-682 and docetaxel significantly slowed tumor growth (Fig. 4h-i).

## SX-682 increases susceptibility to docetaxel in the murine MOC1 HNSCC model

**Fig. 5:**
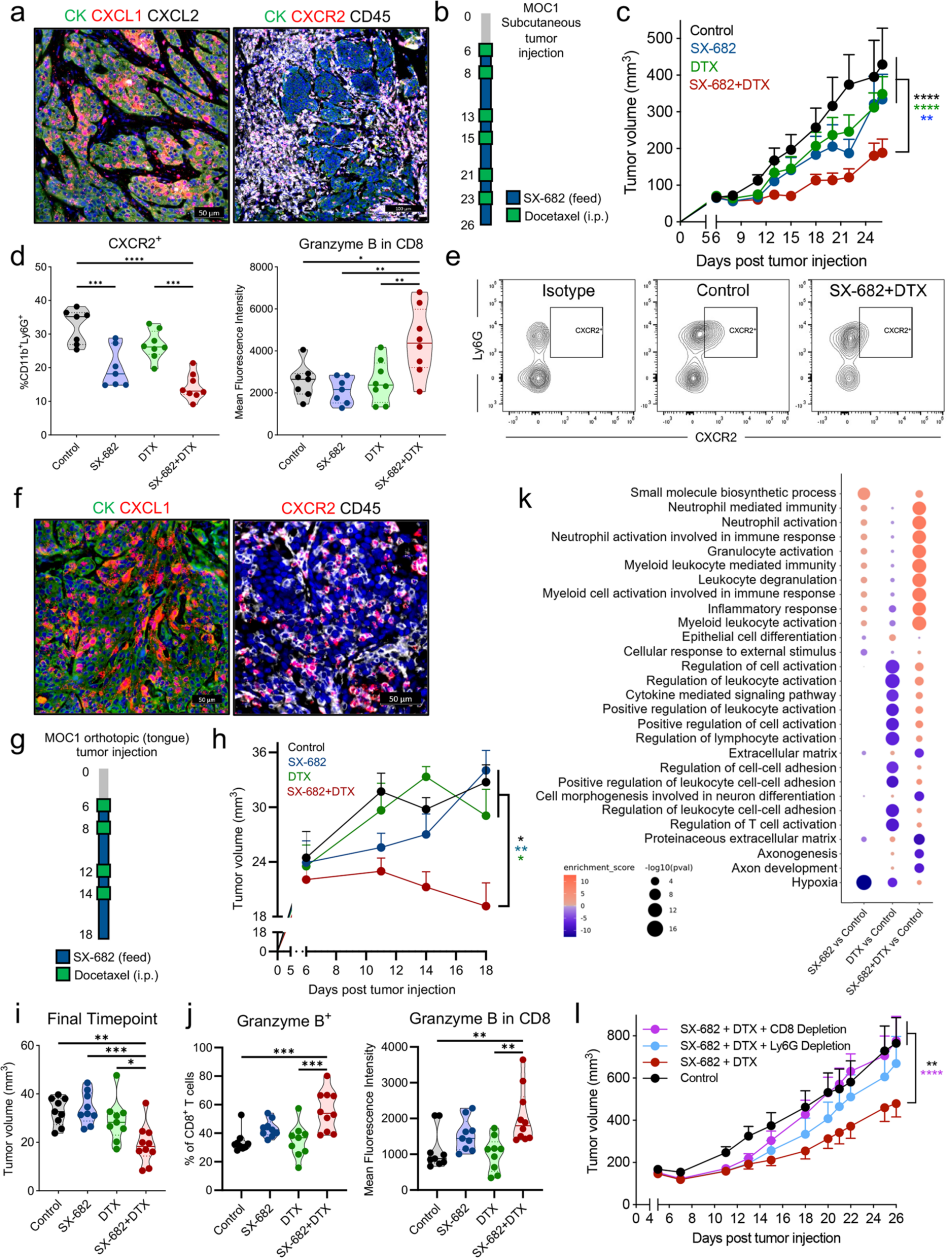
CXCR1/2 inhibition increases susceptibility of murine HNSCC models to docetaxel leading to enhanced anti-tumor immunity. **a** MOC1 tumors were evaluated via RNA in situ hybridization for the expression of murine IL-8 homologs CXCL1 (red) and CXCL2 (white) or the receptor CXCR2 (red) and immunofluorescence for pan-cytokeratin (CK, green) and CD45 (white). Nuclear staining was performed by DAPI (blue). **b** Schematic depicting the timeline of therapy. Control or SX-682 feed (200 mg/kg) was administered to mice starting on day 6 and remained for the duration of the experiment. Docetaxel (5 mg/kg) was delivered by i.p. injection on indicated days. **c** Average tumor growth (*n* = 7–8 mice/group). Data are representative of 1 of 2 independent experiments. **d** Flow cytometry analysis of indicated immune infiltrating cells in the tumors of MOC1 treated mice. **e** Representative scatter plots depicting the expression of CXCR2 on Ly6G^+^ tumor infiltrating cells in control and SX-682 plus docetaxel groups. **f** Orthotopic MOC1 tumors were evaluated via RNA in situ hybridization for the expression of CXCL1 (red, left) and CXCR2 (red, right) and immunofluorescence for pan-cytokeratin (CK, green) and CD45 (white). Nuclear staining was performed by DAPI (blue). **g** Schematic depicting the timeline of therapy for treatment of orthotopic MOC1 tumors with dosing as in (**b**). **h** Average tumor growth (*n* = 9–10 mice/group). **i** Tumor volumes recorded at the final timepoint of the experiment. **j** Flow cytometry analysis of indicated immune infiltrating cells in the tumors from (**h**, **i**). **k** Bulk RNAseq transcriptome analysis was performed on mRNA isolated from control, SX-682, docetaxel, and SX-682 plus docetaxel treated MOC1 tumors from (**c**); *n* = 5 mice per group. Bubble plots depicting the top activated and deactivated GO and HALLMARK pathways when comparing SX-682, docetaxel, and SX-682 plus docetaxel treated tumors to control tumors are shown. **l** Average tumor growth of MOC1 tumors untreated or treated with SX-682 plus docetaxel with or without administration of depleting antibodies for CD8^+^ or Ly6G.^+^ cells; *n* = 8 mice in control, 9 mice in SX-682 plus docetaxel, and 6 mice in both CD8 and Ly6G depletion groups. Values graphed in (**c**, **h**, **l**) represent the mean ± SEM; * *p* < 0.05, ** *p* < 0.01, *** *p* < 0.001, **** *p* < 0.0001 for 2-way ANOVA in (**c**, **h**, **l**), and for 1-way ANOVA followed by Tukey’s post hoc test in (**d**, **i**, **j**)

To determine if SX-682 could be efficacious in a syngeneic, immunocompetent murine model of HPV-negative HNSCC, MOC1 tumors implanted s.c. in the flank of mice were evaluated via RNA in situ hybridization for expression of CXCR2 and the murine CXCL chemokines, CXCL1 and CXCL2, as mice lack the IL-8 gene (Fig. 5a). Tumor cells, identified as CK positive cells, expressed high levels of CXCL1 and low levels of CXCL2. Unlike the xenograft models, CXCR2 was predominantly expressed in CD45^+^ immune cells with minimal to no expression observed in CK positive MOC1 tumor cells, allowing us to evaluate the effect of SX-682 plus docetaxel on the immune compartment with no direct effect on the tumor cells. MOC1 tumor bearing mice were treated as indicated (Fig. 5b); while neither monotherapy had a significant effect (Fig. 5c), the combination therapy led to a significant delay in tumor growth. Flow cytometry analysis of tumor-infiltrating leukocytes (TIL) collected at the experimental endpoint revealed a significant decrease in CXCR2^+^ CD11b^+^ Ly6G^+^ cells in tumors from mice that received SX-682-medicated feed (Fig. 5d-e), and a significant increase in granzyme B expression in CD8^+^ T cells in the tumors treated with combination therapy compared to all other groups. MOC1 tumors implanted orthotopically in the tongue of mice were also evaluated (Supp. Figure 3). Similar to the s.c. flank model, orthotopic MOC1 tumors exhibited high levels of CXCL1 expression in tumor cells and CXCR2 expression in CD45^+^ immune cells when evaluated by RNA in situ hybridization (Fig. 5f). Combined treatment with SX-682 and docetaxel as indicated (Fig. 5g) led to a significant delay in tumor growth (Fig. 5h-i) compared to control tumors or those treated with each agent as a monotherapy. Flow cytometry analysis of tumor infiltrating immune cells showed increased frequency of CD8^+^ TIL that were granzyme B positive and had greater granzyme B levels compared to all other groups (Fig. 5j). To potentially elucidate immunological mechanisms involved in the anti-tumor activity of the combination SX-682 plus docetaxel, RNA sequencing analysis of whole tumor tissue from individual mice in Fig. 5c was conducted. As shown in Fig. 5k, multiple gene pathways were differentially expressed between tumors in the combination compared to the control group, including activation of inflammatory response pathways (Supp. Figure 4, left), granulocyte activation pathways (Supp. Figure 4 right), and regulation of leukocyte activation pathways. In addition, extracellular matrix pathways were significantly deactivated in the combination group vs. the control. As the transcriptomics data and flow cytometry data both suggested activation of immune cells in tumors from the combination group, we conducted a depletion study to understand the contribution of CD8^+^ T cells and granulocytes to the anti-tumor effect of the combination therapy. As shown in Fig. 5l, depletion of CD8^+^ T cells completely abrogated the antitumor effects of SX-682 plus docetaxel therapy in the MOC1 s.c. model. Interestingly, the depletion of Ly6G^+^ cells modestly impaired the efficacy of the combination therapy albeit without reaching statistical significance. These data indicated that CD8^+^ T cells and, to a lesser extent, granulocytes may be implicated in the anti-tumor effect of CXCR1/2 inhibition plus docetaxel.

## Discussion

This study reports two major mechanistic findings through which CXCR1/2 inhibition plus docetaxel chemotherapy exhibit synergy in models of HPV-negative HNSCC: (a) a tumor cell intrinsic mechanism in which CXCR1/2 inhibition modulates miR-200c and tubulin beta-3 expression to uniquely sensitize cancer cells to the cytotoxic activity of docetaxel, and (b) an immune-mediated mechanism characterized by enhanced T-cell activation and reduced suppressive myeloid cells, ultimately promoting anti-tumor immunity.

The chemokine IL-8 is one of multiple ligands that bind to the CXCR1/2 receptors. IL-8 has been studied as a prognostic marker in many cancer types [11], and high expression of IL-8 in the serum or plasma of cancer patients has been correlated with worse disease prognosis and poor survival, including in patients with breast [28], ovarian [29], and pancreatic cancer [30]. The chemokine receptor CXCR2 has also been shown to be elevated in the stroma and tumor cells of adenocarcinomas and squamous cell carcinomas of the lung, and its overexpression was associated with shorter survival [31]. In the case of head and neck cancers, high levels of IL-8 have been observed in circulation, saliva, or tumor tissues in several studies [32]. For example, a study by Chen et al. [33] reported high concentration of IL-8 in the serum of patients with HNSCC, compared to patients with laryngeal papilloma or age-matched healthy donors, and a similar study showed significantly higher levels of circulating IL-8 in patients with metastatic or local/regional recurrent HNSCC, compared to healthy controls [14]. In both studies, however, no distinction was made regarding HPV-positive and HPV-negative HNSCC cases. In addition to circulating IL-8 levels, high expression of IL-8 has been reported in HNSCC tumor tissues. A recent study evaluated the expression of IL-8 mRNA via RT-PCR in 87 HNSCC tumor biopsies obtained prior to treatment with radiotherapy or chemo-radiotherapy. The study reported significantly higher levels of IL-8 mRNA in tumor tissues compared with healthy mucosa but had no information on HPV status, and showed that patients with high IL-8 mRNA levels had a 5-year local recurrence-free survival of 65.5% compared to 90.2% for those with low IL-8 mRNA in the tumor pre-treatment [34]. In this study, we have shown that IL-8 is predominantly expressed in HPV-negative compared to HPV-positive tumors or cell lines. In addition, the utilization of RNA in situ hybridization for detection of IL-8 mRNA allowed the visualization of the localization of IL-8, which was found mostly on tumor cells with little expression observed in CD45^+^ infiltrating immune cells.

The role of CXCR1/2 signaling in cancer has been primarily investigated in preclinical models regarding its ability to promote the migration of myeloid cells. Overall, high levels of circulating MDSC have been linked to advanced stages of HNSCC [35], and some studies have shown that high frequency of PMN-MDSC in peripheral blood of head and neck cancer patients is associated with poor survival [36]. A study comparing HPV-positive and HPV-negative oropharyngeal squamous cell carcinomas (OPSCC) showed that HPV-negative OPSCC contained higher numbers of neutrophils compared to HPV-positive cases, and that HPV-negative OPSCC tumor-stromal culture models produced higher levels of IL-8 and other chemokines compared to HPV-positive models [37]. These data together with our own findings of higher expression of IL-8, CXCR1, and CXCR2 in HPV-negative HNSCC highlight the potential for CXCR1/2 blockade as a therapeutic strategy in this patient population. In addition, prior work from our laboratory demonstrated that signaling through CXCR1/2 receptors expressed in carcinoma cells enables their acquisition of mesenchymal tumor features which, in turn, can drive resistance to EGFR-targeted therapy in lung cancer models [7] or immune-mediated lysis in models of breast cancer [8]. To evaluate whether blockade of CXCR1/2 receptors could impact responses to chemotherapy in HNSCC, here we used SX-682, a clinical stage, dual small-molecule inhibitor of CXCR1 and CXCR2 that was previously shown with multiple preclinical tumor models to decrease the migration of immune suppressive, CXCR2-positive PMN-MDSC from the periphery into the tumor, leading to improved tumor control [9, 10, 12, 38]. Several clinical trials are currently ongoing evaluating SX-682 in combination with checkpoint inhibition in patients with melanoma, colorectal cancer, pancreatic adenocarcinoma, and non-small cell lung cancer in the metastatic setting. In this study, SX-682 treatment of HPV-negative HNSCC cell line models significantly increased tumor cell susceptibility to docetaxel without affecting responses to cisplatin, which is commonly used as first-line treatment for advanced HNSCC cases. The dose of SX-682 used in this study (5–10 µM) did not exceed the range of drug plasma concentration found in patients treated with SX-682 in an early phase clinical trial [39]. The improved response of HPV-negative HNSCC tumor cells to docetaxel was confirmed in vivo where the combination of docetaxel and SX-682 induced robust tumor control and tumor cures. Interestingly, we also observed synergy of this combination therapy in a low IL-8 producing model of HPV-positive HNSCC in vivo, suggesting the potential application of the approach in a broader context for HNSCC treatment.

In search of the mechanism(s) involved in the sensitization of HNSCC tumor cells to docetaxel, we found that blockade of CXCR1/2 increased the expression of miR-200c, a pleiotropic tumor suppressor known to inhibit tumor growth, increase susceptibility to chemotherapy, and block EMT through its main target, ZEB1 [40]. While the loss of miR-200c has contributed to drug resistance to platinum-based chemotherapies in some tumor models [41], in other models it has been shown to selectively modulate resistance to microtubule-targeting agents by regulating the expression of tubulin beta-3 [24]. Tubulins α and β are proteins that heterodimerize and assemble to form cellular microtubules, which play a major role in cell division, cell motility, and vesicle transport. Among various members of the tubulin protein family, tubulin beta-3 is aberrantly overexpressed in several types of epithelial tumors [42], including head and neck cancer, where conflicting results have been reported regarding the association of tubulin beta-3 with clinical outcome [43, 44]. In addition, expression of tubulin beta-3 has been directly associated with resistance to microtubule-targeting therapies in some cancer types [45–47]. Consistently, our results showed that treatment of HPV-negative HNSCC cell lines with SX-682 increased miR-200c and decreased the expression of tubulin beta-3 in vivo, thus potentially implicating the modulation of tubulin beta-3 in the mechanism of SX-682-mediated sensitization to docetaxel chemotherapy.

While our studies with xenograft models in vitro and in vivo detailed the sensitizing effect of CXCR1/2 inhibition to docetaxel efficacy on tumor cells directly, these models did not allow the interrogation of the effect of these agents on the immune system. Therefore, we utilized the syngeneic MOC1 murine model of HPV-negative HNSCC both subcutaneously and orthotopically to elucidate how CXCR1/2 inhibition plus docetaxel reshapes the tumor immune microenvironment. While MOC1 tumor cells did not express high levels of CXCR1/2, we did observe a population of CXCR2^+^CD11b^+^Ly6G^+^ myeloid cells in the spleens and tumors of these mice which have been shown to be highly immune suppressive [48]. Consistent with previous reports, SX-682 reduced the amount of CXCR2^+^CD11b^+^Ly6G^+^ myeloid cells in the TME, while cytotoxic, granzyme B^+^ CD8^+^ T cells were significantly increased in tumors when SX-682 was used in combination with docetaxel. Depletion of CD8^+^ T cells completely abrogated the anti-tumor effect of the combination therapy in MOC-1 tumor bearing mice, indicating the relevant role of an adaptive immune response in tumor control. Interestingly, depletion of Ly6G^+^ cells, while not statistically significant, also showed a trend towards decreasing anti-tumor efficacy, suggesting that Ly6G^+^ myeloid cells could play a supporting role in the observed anti-tumor efficacy of the combination therapy. In agreement with this hypothesis, the results of bulk RNAseq of tumors in the combination group showed a significant upregulation of genes involved in neutrophil and granulocyte activation pathways in the TME. The biology of neutrophils in the TME is highly diverse [49]. While elimination of suppressive CXCR2^+^ PMN-MDSC can enhance antitumor immunity, many studies have highlighted the critical role of neutrophils to drive antitumor responses [50, 51]. The results of this study, including FACS analysis of myeloid cell populations, RNAseq, and depletion of Ly6G^+^ cells suggest that the combination of CXCR1/2 inhibition and docetaxel treatment may have either altered the balance of suppressive and anti-tumor myeloid cell populations, or promoted the reprogramming of immature, suppressive myeloid populations into a more anti-tumor phenotype. Future studies will explore this hypothesis in various preclinical models, including in several well-validated orthotopic HNSCC models such as AT-84 and 4MOSC1 [52, 53].

Although cisplatin is the most frequently administered chemotherapy for patients with HPV-negative HNSCC, docetaxel is used in several clinical settings, including as definitive treatment in combination with radiation in patients who are ineligible for cisplatin, and in several combination treatments for relapsed disease [54]. Importantly, treatment of relapsed HPV-negative HNSCC patients with pembrolizumab alone or in combination with cisplatin and 5-FU rarely results in cures, creating a significant pool of patients with relapsed disease that have failed first-line treatment. To date, no proven, highly effective second-line treatment options exist for patients who have failed to respond to pembrolizumab.

## Conclusions

The results of this study indicate that inhibition of the chemokine receptors, CXCR1/2, can uniquely sensitize cancer cells to the cytotoxic activity of docetaxel, while the combination of CXCR1/2 inhibition plus docetaxel could also reshape the immune microenvironment in HPV-negative HNSCC to promote anti-tumor immunity. These findings provide a direct rationale for the clinical study of SX-682 or other inhibitors of CXCR1/2 in combination with docetaxel in the second-line treatment of patients with HPV-negative HNSCC who have failed first-line pembrolizumab treatment. The findings also provide rationale for the study of future combinations of CXCR1/2 inhibition, docetaxel, and immune-based therapies.

## Supplementary Information

Supplementary Material 1.

Supplementary Material 2.

## Data Availability

The datasets analyzed for the current study are available from the corresponding author on reasonable request. Bulk RNA-seq data will be made available in NCBI’s Gene Expression Omnibus at the time of publication.

## Abbreviations

HNSCC: Head and neck squamous cell carcinoma
PD-1: Programmed cell death 1 receptor
IL-8: Interleukin-8
PMN-MDSC: Polymorphonuclear myeloid-derived suppressor cells
TME: Tumor microenvironment
EMT: Epithelial-mesenchymal transition
TCGA: The Cancer Genome Atlas
MOC1: Mouse oral cancer-1
ROI: Regions of interest
MFI: Mean fluorescence intensity
NIDAP: NIH Integrated Data Analysis Platform
DEG: Differentially expressed genes
GSEA: Gene set enrichment analysis
ELISA: Enzyme-linked immunosorbent assay
TCM: Tumor-conditioned media
i.p.: Intraperitoneal
CK: Pan-cytokeratin
miR: MicroRNA
TIL: Tumor-infiltrating leukocytes
