# Supplementary material for: Inhibition of the chemokine receptors CXCR1 and CXCR2 synergizes with docetaxel for effective tumor control and remodeling of the immune microenvironment of HPV-negative head and neck cancer models
Source: J Exp Clin Cancer Res. 2024 Dec 5;43:318. doi: 10.1186/s13046-024-03240-3 (PMC11619435; doi:10.1186/s13046-024-03240-3)
Supplement: Supplementary file 1 — Supplementary Material 1. [file 13046_2024_3240_MOESM1_ESM.pdf]

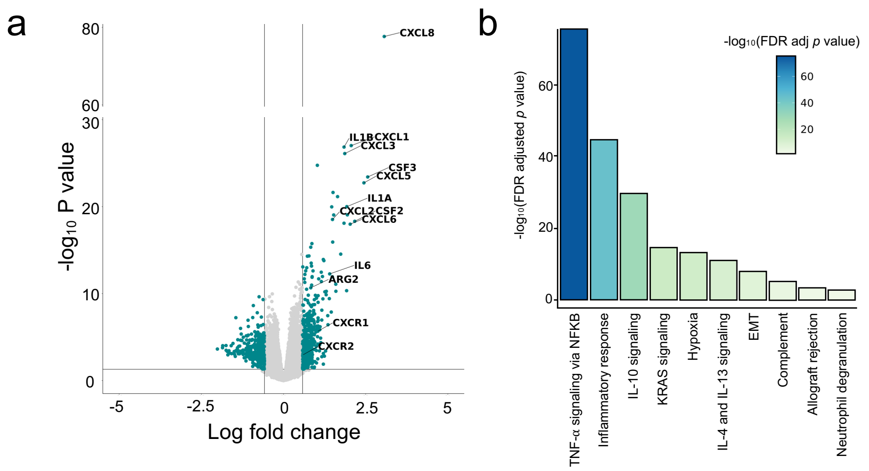

Supplementary Figure 1. (a) Volcano plot of differentially expressed genes between IL-8 high (n=205) and IL-8 low (n=205) expressing HPV-negative tumors in the TCGA head and neck cancer database. (b) Top 10 activated HALLMARK and Reactome pathways when comparing IL-8 high and IL-8 low expressing HPV-negative tumors from (a).

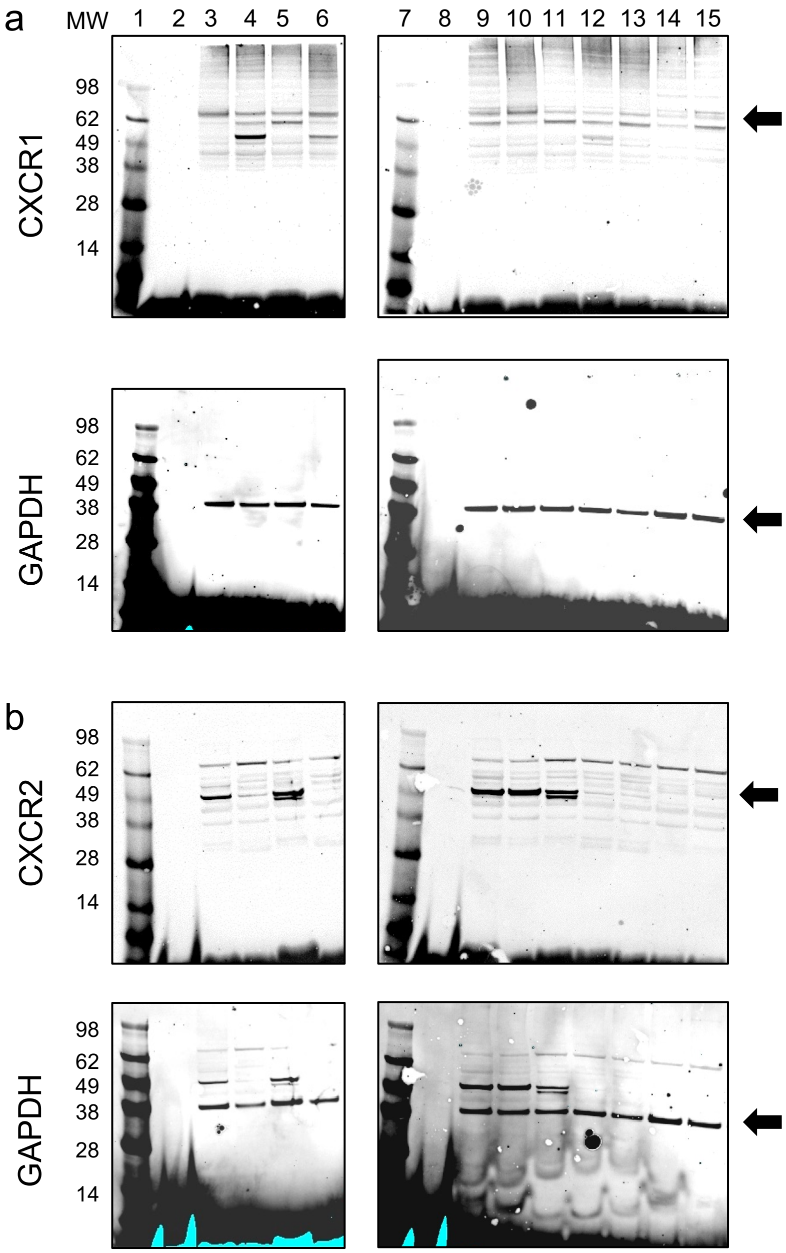

Supplementary Figure 2. Whole immunoblots from Figure 1p. (a,b) Lanes: 1, molecular weight ladder; 2, blank; 3, UPCI-SCC-154; 4, UPCI-SCC-152; 5, UM-SCC-47; 6, UPCI-SCC-90; 7, molecular weight ladder; 8, blank; 9, UM-SCC-1; 10, UM-SCC-11A; 11, UM-SCC-11B; 12, UM-SCC-22A; 13, UM-SCC-22B; 14, UM-SCC-74A; 15, UM-SCC-74B. The same blot was probed with antibodies to detect CXCR1 and GAPDH (a) or CXCR2 and GAPDH (b). MW column indicates the molecular weight of the corresponding ladder band. Black arrows indicate the approximate molecular weight of the band of interest.

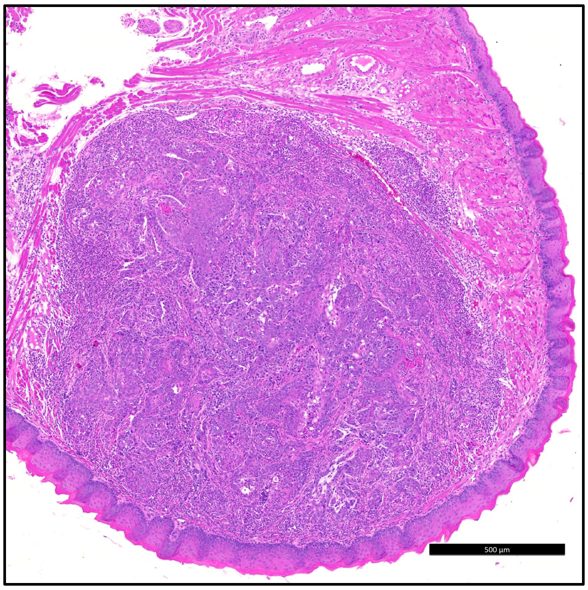

Supplementary Figure 3. Hematoxylin and eosin stain of untreated orthotopic MOC1 tumor in murine tongue.

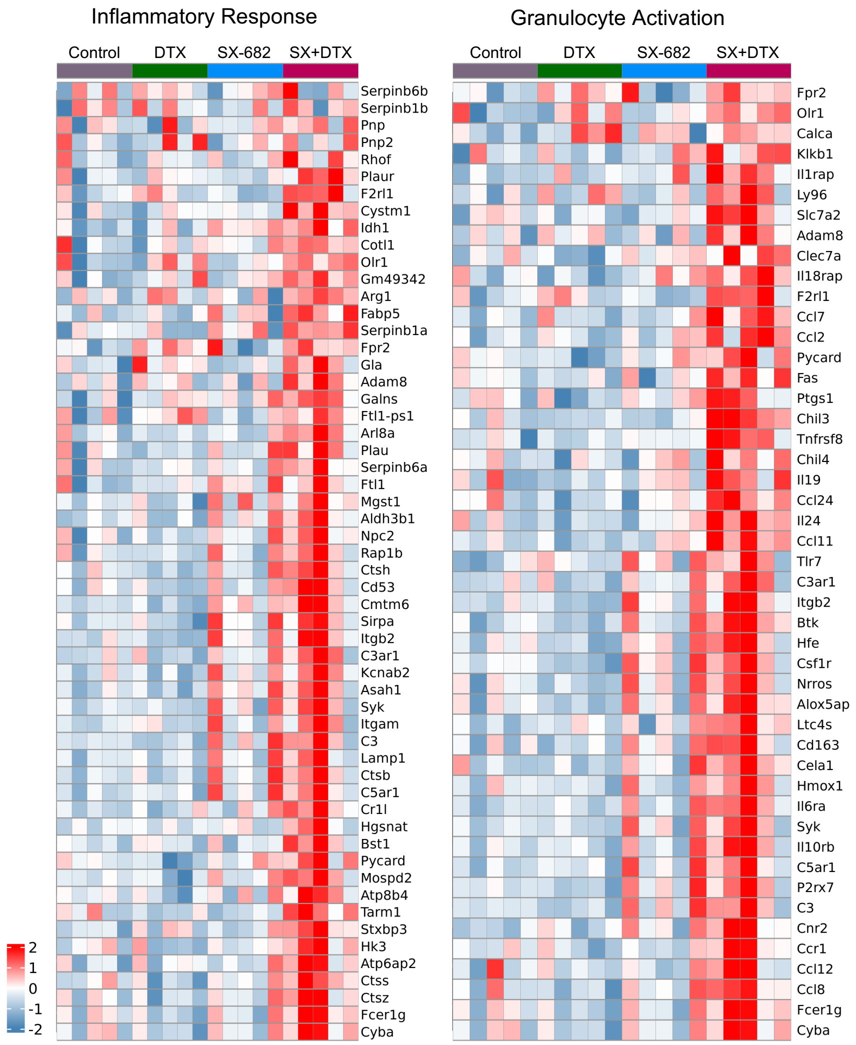

Supplementary Figure 4. Heatmaps of differentially expressed genes between the SX-682 plus docetaxel and control treatment groups in the GO Inflammatory Response (left) and the GO Granulocyte Activation (right) pathways.
