# Supplementary material for: Inhibition of the chemokine receptors CXCR1 and CXCR2 synergizes with docetaxel for effective tumor control and remodeling of the immune microenvironment of HPV-negative head and neck cancer models
Source: J Exp Clin Cancer Res. 2024 Dec 5;43:318. doi: 10.1186/s13046-024-03240-3 (PMC11619435; doi:10.1186/s13046-024-03240-3)
Supplement: Supplementary file 2 — Supplementary Material 2. [file 13046_2024_3240_MOESM2_ESM.pdf]

Supplemental Table 1. Analysis of CXCR1 and CXCR2 expression via multiplex immunofluorescence in head and neck normal and cancer tissues.

No.	Organ	Pathology Diagnosis	Grade	Stage	TNM	Type	CK^{POS} $CXCR1^{POS}$	CK^{NEG} $CXCR1^{POS}$	CK^{POS} $CXCR2^{POS}$	CK^{NEG} $CXCR2^{POS}$
1	Nasopharynx	Squamous cell ca.	1	I	T1N0M0	Malignant	Pos	Pos	Pos	-
2	Nasopharynx	Squamous cell ca.	3	II	T2N0M0	Malignant	Pos	Pos	-	-
3	Left maxillary sinus	Squamous cell ca.	1	I	T1N0M0	Malignant	-	-	-	-
4	Left maxillary sinus	Squamous cell ca.	2	II	T2N0M0	Malignant	Pos	-	Pos	-
5	Maxillary sinus	Squamous cell ca.	2	II	T2N0M0	Malignant	Pos	Pos	Pos	Pos
6	Left maxillary sinus	Squamous cell ca.	3	II	T2N0M0	Malignant	Pos	Pos	Pos	Pos
7	Right septal sinus	Squamous cell ca.	3	II	T2N0M0	Malignant	Pos	-	Pos	-
8	Nasopharynx	Squamous cell ca.	3	II	T2N0M0	Malignant	Pos	Pos	-	-
9	Nasopharynx	Squamous cell ca.	3	II	T2N0M0	Malignant	-	-	-	-
10	Nasopharynx	Squamous cell ca.	3	III	T3N0M0	Malignant	Pos	-	-	-
11	Nasopharynx	Squamous cell ca.	3	III	T3N0M0	Malignant	-	Pos	-	-
12	Oral Cavity	Squamous cell ca.	1	III	T3N0M0	Malignant	Pos	Pos	Pos	-
13	Oral Cavity	Squamous cell ca.	1	III	T3N0M0	Malignant	Pos	Pos	Pos	Pos
14	Oral Cavity	Squamous cell ca.	1	II	T2N0M0	Malignant	-	Pos	Pos	Pos
15	Oral Cavity	Squamous cell ca.	1	IVA	T4aN1M0	Malignant	-	-	-	-
16	Oral Cavity	Squamous cell ca.	2	IVA	T4aN1M0	Malignant	-	-	Pos	-
17	Oral Cavity	Squamous cell ca.	2	III	T3N0M0	Malignant	Pos	Pos	Pos	-
18	Oral Cavity/Soft palate	Squamous cell ca.	1	II	T2N0M0	Malignant	Pos	Pos	Pos	Pos
19	Oral Cavity/Palate	Squamous cell ca.	2	II	T2N0M0	Malignant	-	-	-	-
20	Oral Cavity/Tongue	Squamous cell ca.	1	II	T2N0M0	Malignant	Pos	Pos	Pos	Pos
21	Oral Cavity/Root of tongue	Squamous cell ca.	1	IVA	T4aN0M0	Malignant	Pos	-	Pos	-
22	Oral Cavity/Tongue	Squamous cell ca.	1	II	T2N0M0	Malignant	Pos	-	Pos	-
23	Oral Cavity/Tongue	Squamous cell ca.	1	III	T3N1M0	Malignant	Pos	Pos	Pos	Pos
24	Oral Cavity/Tongue	Squamous cell ca.	1	II	T2N0M0	Malignant	Pos	Pos	Pos	Pos
25	Oral Cavity/Tongue	Squamous cell ca.	1	II	T2N0M0	Malignant	Pos	Pos	Pos	Pos
26	Oral Cavity/Tongue	Squamous cell ca.	1	II	T2N0M0	Malignant	Pos	Pos	Pos	-
27	Oral Cavity/Tongue	Squamous cell ca.	1	II	T2N0M0	Malignant	Pos	Pos	Pos	Pos
28	Oral Cavity/Tongue	Squamous cell ca.	1	II	T2N0M0	Malignant	Pos	Pos	Pos	Pos
29	Oral Cavity/Tongue	Squamous cell ca.	1	II	T2N0M0	Malignant	Pos	Pos	Pos	Pos
30	Oral Cavity/Tongue	Squamous cell ca.	1	III	T3N1M0	Malignant	Pos	Pos	Pos	Pos
31	Oral Cavity/Tongue	Squamous cell ca.	1	III	T2N1M0	Malignant	-	-	Pos	Pos
32	Oral Cavity/Tongue	Squamous cell ca.	1	III	T2N1M0	Malignant	-	-	Pos	Pos
33	Oral Cavity/Tongue	Squamous cell ca.	2	II	T2N0M0	Malignant	-	Pos	-	-
34	Oral Cavity/Tongue	Squamous cell ca.	2	II	T2N0M0	Malignant	Pos	Pos	Pos	Pos
35	Oral Cavity/Tongue	Squamous cell ca.	2	III	T3N1M0	Malignant	Pos	Pos	Pos	Pos
36	Oral Cavity/Tongue	Squamous cell ca.	2	II	T2N0M0	Malignant	Pos	Pos	Pos	Pos
37	Oral Cavity/Tongue	Squamous cell ca.	3	III	T3N0M0	Malignant	-	-	-	-
38	Oral Cavity/Gingiva	Squamous cell ca.	1	II	T2N0M0	Malignant	-	-	-	-
39	Oral Cavity/Gingiva	Squamous cell ca.	1	II	T2N0M0	Malignant	Pos	Pos	Pos	-
40	Oral Cavity/Gingiva	Squamous cell ca.	1	II	T2N0M0	Malignant	-	-	Pos	-

No.	Organ	Pathology Diagnosis	Grade	Stage	TNM	Type	CK^{POS} $CXCR1^{POS}$	CK^{NEG} $CXCR1^{POS}$	CK^{POS} $CXCR2^{POS}$	CK^{NEG} $CXCR2^{POS}$
41	Oral Cavity/Gingiva	Squamous cell ca.	2	II	T2N0M0	Malignant	Pos	-	Pos	-
42	Oral Cavity/Gingiva	Squamous cell ca.	2	III	T3N0M0	Malignant	-	-	Pos	-
43	Oral Cavity/Gingiva	Squamous cell ca.	2	II	T2N0M0	Malignant	Pos	-	Pos	-
44	Oral Cavity/Gingiva	Squamous cell ca.	2	III	T3N1M0	Malignant	-	-	-	-
45	Oral Cavity/Gingiva	Squamous cell ca.	2	II	T2N0M0	Malignant	-	-	-	-
46	Oral Cavity/Gingiva	Squamous cell ca.	2	II	T2N0M0	Malignant	-	-	-	-
47	Tonsil	Squamous cell ca.	1	II	T2N0M0	Malignant	-	-	-	-
48	Tonsil	Squamous cell ca.	2	II	T2N0M0	Malignant	-	Pos	Pos	-
49	Tonsil	Squamous cell ca.	3	II	T2N0M0	Malignant	-	-	-	-
50	Oropharynx	Squamous cell ca.	1	III	T1N1M0	Malignant	Pos	Pos	Pos	-
51	Oropharynx	Squamous cell ca.	1	II	T2N0M0	Malignant	-	-	-	-
52	Oropharynx	Squamous cell ca.	2	II	T2N0M0	Malignant	Pos	Pos	-	-
53	Oropharynx	Squamous cell ca.	2	II	T2N0M0	Malignant	-	Pos	-	-
54	Oropharynx	Squamous cell ca.	2	IVA	T4aN0M0	Malignant	-	-	Pos	-
55	Oropharynx	Squamous cell ca.	2	III	T2N1M0	Malignant	-	Pos	Pos	Pos
56	Oropharynx	Squamous cell ca.	3	III	T2N1M0	Malignant	-	-	Pos	-
57	Oropharynx	Squamous cell ca.	3	II	T2N0M0	Malignant	-	-	-	-
58	Epiglottis	Squamous cell ca.	2	III	T3N1M0	Malignant	-	-	Pos	-
59	Epiglottis	Squamous cell ca.	2	II	T2N0M0	Malignant	Pos	-	Pos	-
60	Oropharynx	Squamous cell ca.	2	II	T2N0M0	Malignant	Pos	Pos	Pos	Pos
61	Oropharynx	Squamous cell ca.	2	III	T3N0M0	Malignant	-	-	-	-
62	Oropharynx	Squamous cell ca.	2	III	T3N0M0	Malignant	Pos	Pos	Pos	-
63	Laryngopharynx	Squamous cell ca.	2	III	T2N1M0	Malignant	Pos	Pos	-	-
64	Laryngopharynx	Squamous cell ca.	2	II	T2N0M0	Malignant	Pos	Pos	Pos	-
65	Laryngopharynx	Squamous cell ca.	2	II	T2N0M0	Malignant	-	-	-	-
66	Laryngopharynx	Squamous cell ca.	2	IVA	T4aN0M0	Malignant	Pos	Pos	Pos	-
67	Laryngopharynx	Squamous cell ca.	2	III	T2N1M0	Malignant	Pos	Pos	Pos	-
68	Laryngopharynx	Squamous cell ca.	2	II	T2N0M0	Malignant	Pos	Pos	Pos	Pos
69	Laryngopharynx	Squamous cell ca.	2	III	T2N1M0	Malignant	Pos	Pos	Pos	Pos
70	Laryngopharynx	Squamous cell ca.	2	II	T2N0M0	Malignant	Pos	Pos	Pos	Pos
71	Larynx/Supraglottic type	Squamous cell ca.	1	IVA	T4aN0M0	Malignant	Pos	Pos	Pos	-
72	Larynx/Supraglottic type	Squamous cell ca.	2	III	T3N0M0	Malignant	Pos	Pos	Pos	Pos
73	Larynx/Glottic type	Squamous cell ca.	2	II	T2N0M0	Malignant	Pos	Pos	Pos	-
74	Larynx/Lower sanctum type	Squamous cell ca.	2	III	T3N0M0	Malignant	-	-	Pos	-
75	Larynx/Glottic type	Squamous cell ca.	2	IVA	T4aN0M0	Malignant	-	-	-	-
76	Larynx/Lower sanctum type	Squamous cell ca.	2	IVA	T4aN0M0	Malignant	Pos	Pos	Pos	Pos
77	Larynx/Glottic type	Squamous cell ca.	2	II	T2N0M0	Malignant	Pos	Pos	Pos	Pos
78	Larynx/Glottic type	Squamous cell ca.	2	III	T3N1M0	Malignant	Pos	-	Pos	-
79	Epiglottis	Epiglottis tissue	-	-	-	Normal	Pos	Pos	Pos	Pos
80	Oropharynx	Oropharynx tissue	-	-	-	Normal	Pos	Pos	-	-
81	Oral Cavity/Tongue	Tongue tissue	-	-	-	Normal	-	Pos	-	-

Pos, positive; (-), negative.
